# Atlas of human gut-associated lymphoid tissue reveals immunomodulatory interactions of B cells

**DOI:** 10.1126/sciimmunol.ady8948

**Published:** 2026-06-05

**Authors:** Michael J. Pitcher, Xiaowen Sun, Chiara Dionisi, Lucia Montorsi, Sherine H. Kottoor, Jacqueline H.Y. Siu, Roman Laddach, Rosamond Nuamah, Gavin J. Pettigrew, Richard J. Ellis, Cynthia Bishop, Jahangir Sufi, Pawan Dhami, Heli Vaikkinen, Audrey Kelly, Anna Vossenkamper, Deena L. Gibbons, Jo Spencer

**Affiliations:** 1 School of Immunology and Microbial Sciences, King’s College London; London, UK; 2 The Kennedy Institute of Rheumatology, University of Oxford; Oxford, UK; 3 School of Cancer and Pharmaceutical Sciences, King’s College London; London, UK; 4 Department of Surgery, University of Cambridge; Cambridge UK; 5 Advanced Cytometry Platform, Guy’s and St Thomas’ NHS Foundation Trust; London, UK; 6 Genomics Research Platform and Single Cell Laboratory at Guy’s and St Thomas’ NHS Foundation Trust; London, UK; 7 Blizard Institute, Barts and the London School of Medicine and Dentistry, Queen Mary University London; London, UK

## Abstract

Gut-associated lymphoid tissue (GALT) is organized lymphoid tissue that responds chronically to antigens, including whole bacteria, sampled from the gut lumen. The ensuing immunoglobulin A (IgA) plasma cell response disseminates to regulate bacterial populations and to mediate intestinal immune homeostasis. GALT has roles in the development of the innate-like marginal zone B cell population and is also associated with a B cell–mediated contribution to ulcerative colitis (UC) severity and response to therapy. Applying integrated multiomics methodologies, we identified key spatially resolved interactions of B cell subsets including broad regulatory features of double negative 2 (DN2) B cells with potential to maintain homeostasis within microbe-rich mucosa. By contrast, GALT in UC is distorted in composition and spatial distribution of B cell subsets that have altered immunomodulatory potential compared to healthy GALT. Thus, we identify interactions of strategically located B cells as mediators of immunological equilibrium in human gut.

## Introduction

Gut-associated lymphoid tissue (GALT) is active organized lymphoid tissue situated at the boundary between the intestinal microbiota and the host (1). Microbial antigens, including bacteria, are sampled from the gut lumen by the follicle associated epithelium (FAE) that overlies GALT and delivered to the subepithelial dome (SED) below (2–5). Ensuing B cell responses, both T cell–dependent and T cell–independent, can propagate the IgA plasma cell response that migrates from GALT to colonize the extensive lamina propria (LP) (6–8). The IgA secreted by LP plasma cells is actively transported across the intestinal epithelium where it maintains a healthy microbiota composition (9).

In addition to the generation of IgA plasma cells (10, 11), further functions of GALT have been identified including a role in supporting the maturation of innate-like marginal zone B cells (MZB) (12). Mature MZB, that circulate in the blood and locate mostly in the microanatomically defined marginal zone of the spleen, recognize antigens with repeating subunit structures and thus are protective against infections with bacteria that have repeating subunit antigens on their surface such as capsular polysaccharides (13). Some MZB that respond to pneumococcal vaccine have B cell receptors (BCRs) that also have specificity for antigens expressed on gut bacteria (12). Thus, the roles of GALT involve encountering and responding to microbial antigens. Yet, despite being rich in innate ligands, sampled microbes do not drive acute inflammation in the GALT microenvironment (1, 14).

To understand the interactions of B cells in GALT, we studied human appendix that contains abundant well-orientated GALT. Data were acquired using the CosMx platform and segmented single cells were aligned to different cell lineages. By integration with CITE-seq reference data, B cells belonging to different subsets were localized in GALT and imputed gene expression used to enrich the information provided by the CosMx platform. This allowed for exploration of B cell subset interactions with other lineages based on cell proximities. We observed that the SED was the zone with highest B cell interaction strengths. Double negative 2 (DN2) cells were abundant in the SED and had strong cellular interactomes that were mostly immunosuppressive (4). Interactions involving CD86, progranulin and Epstein-Barr virus induced gene 3 (EBI3) expressed by DN2 cells were validated by RNAscope and protein expression in the SED confirmed.

The appendix has been associated with the inflammatory bowel disease ulcerative colitis (UC) in several ways (15, 16). Peri-appendiceal ‘red patches’ can be observed endoscopically around the appendiceal orifice in UC, even when the inflammation is restricted to the distal colon and rectum (17). It has been proposed that appendectomy in early life can be protective against subsequent development of UC, though this is debated (15, 16). Most recently, studies of the cellular content of GALT in UC identified variation associated with clinical response to the drug vedolizumab that blocks interactions between integrin a4b7 and its endothelial ligand mucosal addressin cell adhesion molecule 1 (MAdCAM1) (18). We observed that although B cell follicles are present in appendix in UC, the microanatomy is disrupted including altered distribution of DN2 cells and expression of immunomodulators.

Thus, the maintenance of a chronically active but uninflamed microenvironment in GALT includes the production of immunomodulators by B cells, the disruption of which may be involved with inflammatory processes in the gut.

## Results

### Spatial transcriptomics enables visualization of cell lineages in GALT

**Fig 1:**
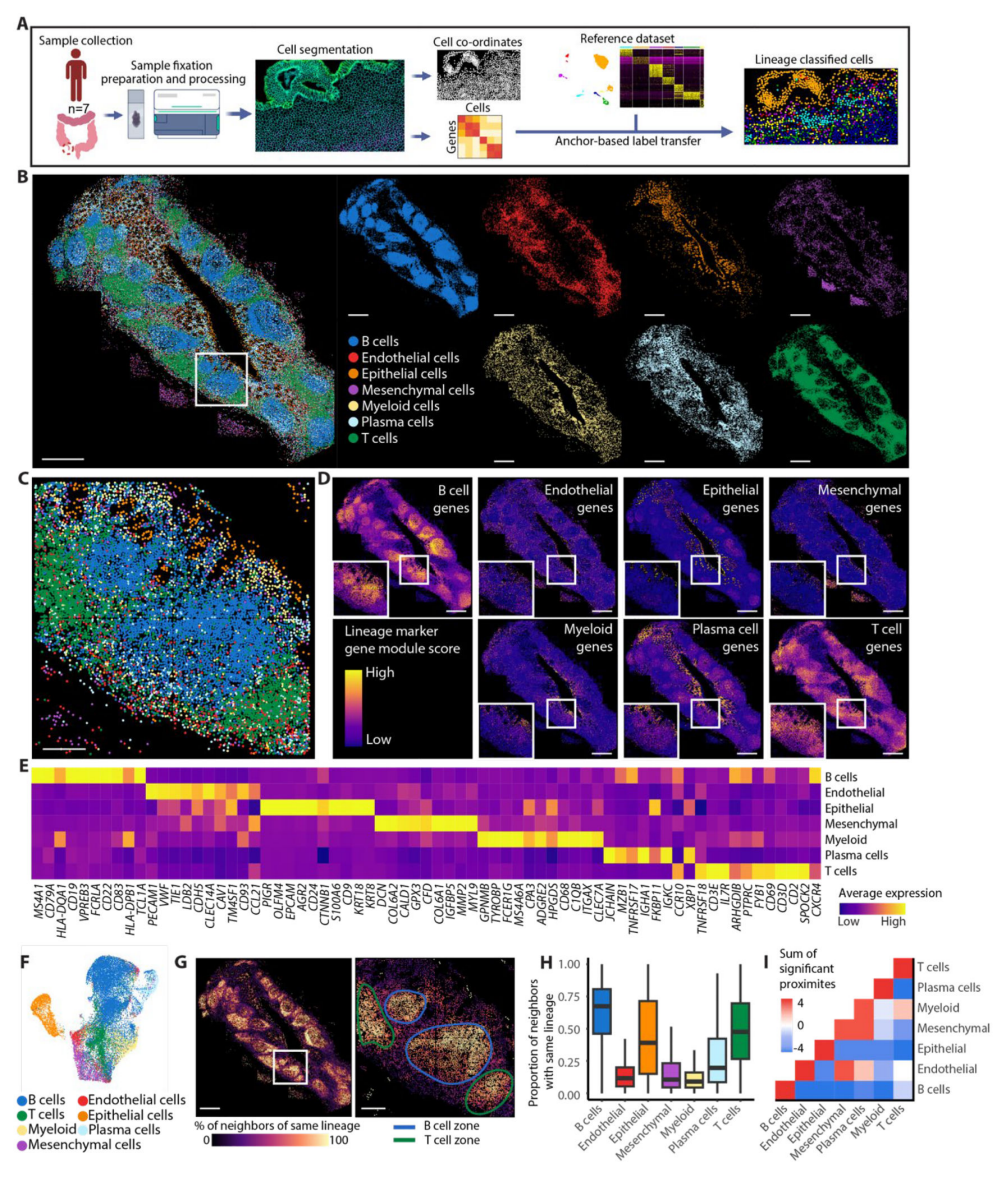
Spatial composition of immune cell lineages in GALT derived using CosMx spatial transcriptomics. (**A**) Schematic of CosMx cell lineage classification. Appendix samples (*n* = 7) were processed using CosMx Spatial Molecular Imager. Processed cell expression data was anchored to a published atlas (19) of immune cells in human appendix prelabeled for cell lineages based on transcriptomic similarity between cells in both datasets. Lineage labels were transferred from reference to CosMx data based on the anchors. (**B**) Spatial organization of cell lineages in appendix visualized spatially (left) in one of the CosMx samples and (right) split by each lineage. (**C**) Spatial lineage classification of a lymphoid follicle from (B) shown in the white box. (**D**) Expression of module scores of marker genes for each lineage derived from annotated reference data, on the whole tissue and inset in (B) and (C) respectively. (**E**) CosMx gene expression heatmap showing top 10 markers for each lineage. (**F**) UMAP visualization of lineage classified cells for a representative sample of the CosMx data since samples were processed individually. (**G**) Percentage of cell neighbors that are the same lineage as the cell of interest (left) across the whole tissue and (right) a lymphoid follicle showing the same region as (C). Regions consisting mostly of T cells (green) and B cells (blue) were manually annotated. (**H**) Boxplot of proportion of neighbors of the same lineage, split by lineage, from all samples combined (means ± SD). (**I**) Heatmap of sum of significant proximities across lineages. *P* values generated by comparing observed with permuted interactions. Positive sum values indicate a tendency for lineages to be in proximity to each other across samples and negative values indicate a tendency for lineages to not be near each other. Schematic created with BioRender (75). Scale bars, [(B), (D), and (G) (left)] 500μm and [(C) and (G) (right)] 100μm.

The aim of this study was to identify cellular interactions of B cells in GALT microenvironments and to identify cell lineages that could contribute to the interactome in data acquired by CosMx (Fig. 1A). After quality control (fig. S1, A and B), segmented cells from the CosMx output were clustered based on their transcripts (fig. S1C). Clusters of cells representing epithelial cells were identified and classified (fig. S1, D and E). Previously published single cell RNA sequencing (scRNA-seq) data acquired from B cells, plasma cells, T cells, myeloid cells, endothelial cells and mesenchymal cells from adult human appendix were used for further lineage classification (19). A weighted anchor-based data transfer method (20) was used to transfer cell lineage labels from the reference dataset to the query CosMx dataset based on transcriptomic similarity between the query CosMx cells and the reference scRNA-seq cells (Fig. 1A). The lineage classifications were then visualized spatially (Fig. 1, B and C and fig. S1F) and validated on the images by comparison with module scores of lineage marker genes (Fig. 1D and data file S1) and average expression of lineage markers per classification (Fig. 1E). Clusters broadly aligned with single lineages (Fig. 1F and fig. S1C), though endothelial cells did not cluster together. Samples displayed similar proportions of cell lineages (fig. S1G).

To compute the relative spatial distribution of cell lineages within the tissues, a neighbor graph was constructed with edges indicating which cells were adjacent to each other with potential for interaction (fig. S1H). Having identified neighboring cells, a metric of neighborhood similarity was calculated for each cell by comparing the proportion of neighboring cells which shared the same lineage as the target cell. When viewed spatially, this metric highlighted large regions of same-lineage interactions (Fig. 1G and fig. S1I). B cells and T cells had the highest neighborhood similarity, illustrating their tendency to reside in lineage-homotypic zones, whereas myeloid cells had the lowest metric, indicating a propensity to reside in regions comprised of different lineages (Fig. 1H). Statistical testing of cell lineage proximities found that all lineages were significantly in proximity to themselves across the samples (Fig. 1I). Mesenchymal cells showed positive interactions with myeloid and endothelial cells. B cells showed negative interactions with all other lineages, further confirming the tendency of most B cells to reside in zones comprised of mostly B cells. Thus, lineage information was assigned to segmented cells in CosMx data to generate profiles consistent with published work, revealing the organized spatial architecture of diverse immune populations in GALT (4, 19, 21).

### Data integration enables B cell subset identification in CosMx images

**Fig 2:**
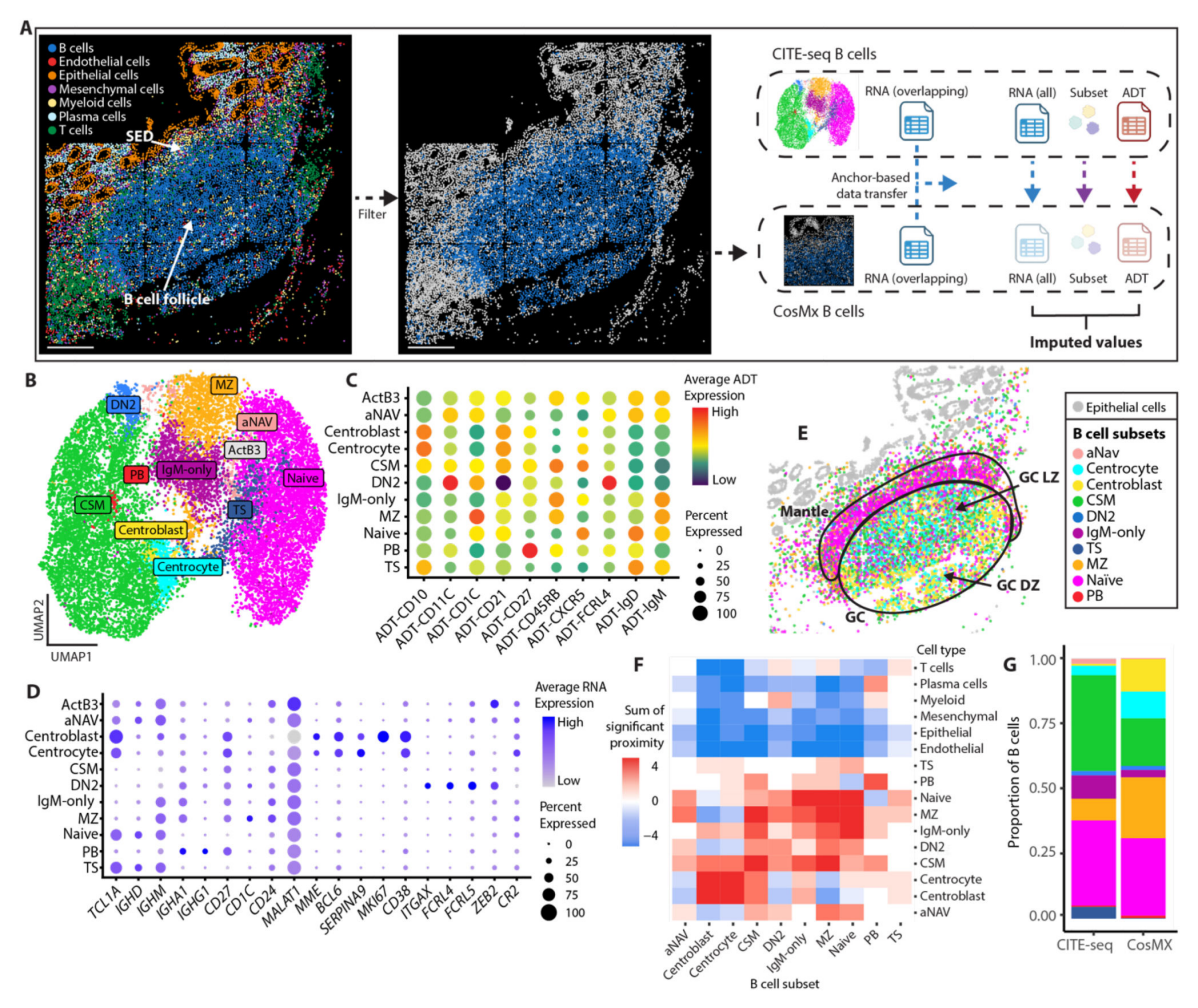
Single-cell analysis of B cell subpopulations in human GALT. (A) Schematic representation of pipeline. B cells were selected from the total CosMx cellular output. A CITE-seq dataset was generated from human appendix cells (*n* = 3) that included RNA sequencing data and antibody-defined tag (ADT) markers enabling B cell subset designation. Common genes between the cells in the CosMx and CITE-seq dataset allowed transfer of cell subset information from CITE-seq to CosMx. Scale bars, 100 μm. (**B**) UMAP visualization of B cell composition in GALT colored by subset (data from *n* = 3 donors). (**C**) Dot plot of surface antibody expression of ADT markers on B cell subsets. (**D**) Dot plot of gene expression of selected genes on B cell subsets. (**E**) A lymphoid follicle from a representative CosMx sample is shown, with manual annotation of the B cell follicle, including the mantle zone of naïve B cells, the germinal center (GC) light zone (LZ) and dark zone (DZ) indicated by arrows. B cell subsets and epithelium indicated by color. (**F**) Heatmap of sum of significant proximities across B cell subsets with cell lineages and B cell subsets. Positive sum values indicate a tendency for cell lineages to be in proximity to each other across samples and negative values indicate a tendency for cell lineages to not be in proximity to each other. (**G**) Comparison of frequencies of B cell subsets observed using CITE-seq and CosMx modalities with the same color code as E. TS, transitional; aNAV, activated naïve; ActB3, activated B cell 3; MZ, marginal zone; CSM, class-switched memory; DN2, double negative 2; PB, plasmablast.

Human GALT contains many potentially interacting B cell subsets, the features which are described in data file S2. To identify the spatial distribution of B cell subsets in GALT within the CosMx data, the lineage-delineated CosMx data was initially filtered down to cells classified as B cells (Fig. 2A). The number and identity of the genes in the 1000-probe set used for CosMx data acquisition were not sufficient to identify the locations of B cells of each subset in the CosMx data. Therefore, to identify B cell subsets, a CITE-seq dataset was generated to allow transfer of B cell subset information by anchor-based data transfer (Fig. 2A)

B cells were sorted from three biological replicates of human appendix, labelled with oligonucleotide tagged antibodies for an antibody detection tag (ADT) (table S1), and loaded into the controller for 10x Chromium 5’ for single cell CITE-seq immune profiling. Gene expression and antibody detection tag (ADT) libraries were prepared and sequenced. After quality control (fig. S2A), data were integrated for both the RNA and ADT assays separately to remove batch effects in each assay. Dimensionality reduction was performed using a weighted-nearest-neighbor approach, combining both the transcriptomic and proteomic data. From the resulting uniform manifold approximation and projection (UMAP), donor of origin was visualized to ensure cells did not separate due to sample (fig. S2B), and the expression of cell surface antibody markers was visualized on the UMAP (fig. S2C). A clustering algorithm assigned cells into 30 distinct clusters based on similarities within both the transcriptome and proteome (fig. S2D). The mean expression of all surface markers (fig. S2E) and a selection of B cell subset–defining transcripts by reference to published work (fig. S2F) were visualized. Where necessary, clusters that showed heterogeneous profiles were split into subclusters using the same method in order to provide greater resolution (fig. S2, D to F). Clusters and subclusters displaying similar profiles were merged and assigned B cell subset labels according to known markers. From within the larger pool of naïve cells, transitional cells were distinguished by their surface expression of CD10 (22), whereas activated naïve (aNAV) were differentiated by surface expression of CD11c and gene expression of Fc receptor-like 5 (*FCRL5*). Germinal center (GC) cells were divided by Ki67 gene expression into centroblasts (Ki67^+^) and centrocytes (Ki67^−^) (23). Centroblasts showed a greater proportion of cells in the G2M phase of the cell cycle (fig. S2G). A DN2 subset of B cells was identified by low surface expression of CD27, IgD, C-X-C Motif Chemokine Receptor 5 (CXCR5) and CD21 and high CD11c surface expression (24). This subset also showed high surface expression of FCRL4 and high gene expression of *FCRL4* and *FCRL5* (25). A subset of activated B cells (ActB3), previously identified in human tissue and blood samples was identified here by high metastasis associated lung adenocarcinoma transcript 1 (*MALAT1*) gene expression (26). MZB were identified by high CD27 and IgM surface expression, whereas IgM-only cells were distinguished from MZB by lower expression of CD1c and IgD and visualized on the UMAP (Fig. 2, B and C) (27, 28). Class switched memory B cells were CD27^+^ and expressed immunoglobulin isotypes other than IgM and IgD (29). The average expression profile for each subset was visualized for both the surface markers (Fig. 2C) and genes (Fig. 2D and data file S1). By linking the spatial CosMx dataset to the single-cell CITE-seq data, ADT marker expression (fig. S3A), B cell subset labels (Fig. 2E) and a higher-depth RNA assay were imputed in the spatial data, allowing identification of spatial densities of B cells subsets (fig. S3B). Statistical tests were applied to B cell subsets to identify significant proximities between B cell subsets and cells of other lineages (Fig. 2F). The only strong positive proximities observed were plasmablasts with plasma cells to which they are developmentally related (30), and DN2 B cells with myeloid cells that have previously been observed to interact in the SED region (4). We compared the representation of each subset within the CITE-seq and CosMx data (Fig. 2G). GC cells were relatively more abundant in the CosMx data. Despite the relative depletion in the cell suspensions, the centroblasts and centrocytes that have known microanatomical compartments (31) were well positioned in the images relative to each other and other cell types following anchor-based transfer of information. Thus, the integration of data across platforms enables analysis of B cell subset interactions and their spatial distribution.

### Cellular interactions of B cells in GALT identified within the CosMx dataset

**Fig 3:**
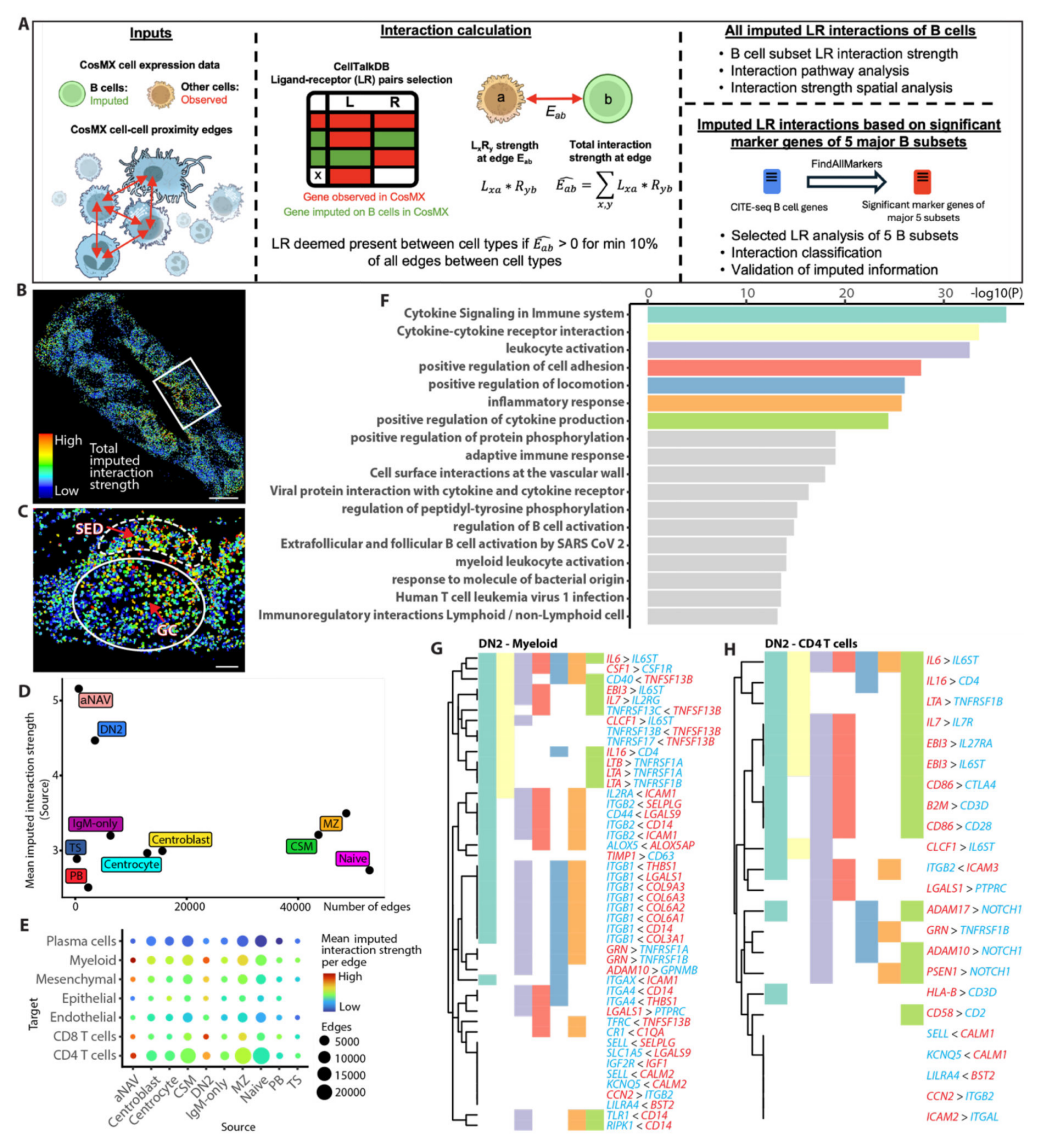
Interaction profile of B cells in GALT derived using ligand-receptor analysis. (**A**) Diagram showing derivation of ligand-receptor interactions from CosMx data and the pipelines leading to outputs in (top) Figs. 3 and (bottom) Fig. 4. (**B**) Ligand-receptor strength between B cells and interacting cells at each edge visualized spatially. Edge positions are mid-point of edge between two cells. Strengths are calculate based on sum of all strengths of ligand-receptor pairs for each edge. (**C**) Expression across a single follicle with manually annotated sub-epithelial dome (SED) identified by dotted line and red arrow and germinal center (GC) identified by solid line and red arrow. (**D**) Scatter plot showing interaction profile of B cell subsets, showing number of edges (horizontal axis) and mean interaction strength (vertical axis). (**E**) Dot plot for B cell subset interactions with B cells as source (expressing ligand). (**F**) Results of pathway analysis run on genes present in the imputed interactions of DN2 B cells in CosMx data. Top seven pathways (with negative log10 *P* value > 20) are highlighted in color. (**G** and **H**) Ligand-receptor pairs present on DN2 interactions with (G) myeloid cells and (H) CD4 T cells. Scale bars, (B) 500 μm and (C) 100 μm.

Having identified the locations of individual cells belonging to major lineages (Fig. 1) and B cells belonging to different subsets in the CosMx data (Fig. 2), we sought to identify interactions between B cells in each subset and neighboring cells of other lineages, in which the B cells could be the target or the source of the interacting factor (Fig. 3A). To achieve this, a reference database of potential “ligand and receptor” gene pairs was used to identify when cell partners within an interacting receptor-ligand pair were present across an edge (32). Here, we defined an interaction as an edge between two cells where one cell expressed a receptor for a ligand expressed by the cell at the other end of the edge. Imputed genes were those expressed by B cells in a subset (data file S1). To be accepted as an interaction, we stipulated that the ligand-receptor pair must involve at least 10% of the edges between the cell types (Fig. 3A).

When interactions were identified, interaction strengths were determined by factoring together normalized reads of the source at one cell on the edge and the target on the opposite cell of the edge (Fig. 3A and data file S3). The combined strength of all target and source pairs for each edge was then visualized, and showed that the SED was associated with the highest interaction strength across the images (Fig. 3, B and C).

Of the B cell subsets, aNAV and DN2 showed the greatest interaction strengths with non-B cell lineages, but with relatively few edges compared to the subsets containing more cells (Fig. 3D and fig S4A). This is consistent with the biased distribution of DN2 towards the SED and FAE that contain many cell types (4).

The strengths of interactions between B cell subsets and cells of other lineages were then determined. Strong interactions of DN2 cells with CD4 T cells, CD8 T cells, mesenchymal and myeloid cells were observed, where DN2 cells were the source cells or the targets of the interaction (Fig. 3E and fig. S4B). After determining imputed interactions between B cell subsets and other lineages, the genes imputed on the DN2 cells that were involved in cellular interactions were run through pathway analysis to determine associated cell function. This identified that the interactions of DN2 B cells were broadly associated with cytokine signaling, leukocyte activation, cell trafficking and cell migration (Fig. 3F). Interactions between DN2 B cells and myeloid cells (Fig. 3G) CD4 T cells (Fig. 3H) and CD8 T cells (fig. S4C) were identified and grouped according to pathways identified in Fig. 3F.

This initial screen of interactions of B cells in different subsets identified some interactions that were not driven by significant features of the B cell subset (data file S1). For example, *IL16* was identified as a mediator of interactions between DN2 B cells and CD4 expressing myeloid and T cells (Fig. 3, G and H), yet *IL16* is not a marker gene of DN2 B cells (data file S1). Using an imaging mass cytometry (IMC) panel that incorporated an RNAscope probe targeting the gene encoding *IL16* (data file S4), we determined that *IL16* was expressed by most B cells (fig. S4, D to G), and that the interaction was likely due to the microenvironmental proximity of DN2 cells to CD4^+^ T cells and myeloid cells in the SED (fig. S4, F and G). Thus, B cell–derived IL16 interacting with the CD4 molecule may have functional relevance in tissues, but this is not an interaction specifically associated with any B cell subset.

### B cell subset–specific markers are involved in cell-cell interactions

**Fig 4:**
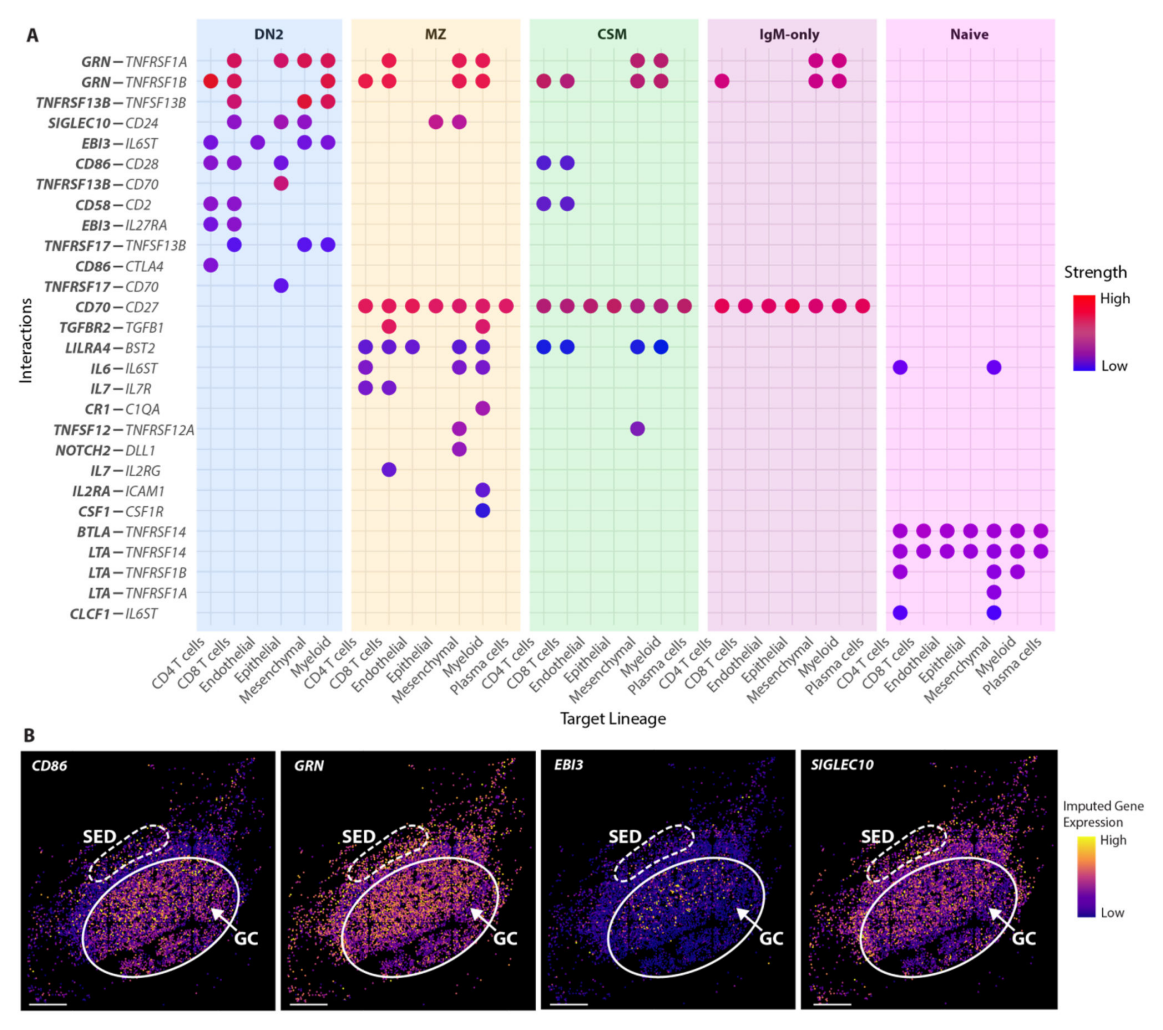
Interactions of five B cell subsets in GALT with cells of other lineages. (**A**) Interaction strengths between marker genes of five B cell subsets identified in the CITE-seq data (*n* = 3) based on sum of all strengths of ligand-receptor pairs for each edge and target cells of other lineages in the CosMx data (*n* = 7) as described in Fig. 3A. Interacting pairs are ranked according to the strength of interactions observed between DN2 B cells and cells of other lineages. (**B**) Spatial analysis of imputed expression of *CD86, GRN, EBI3*, and *SIGLEC10* that are associated with DN2 B cell interactions. Each is enriched in the sub-epithelial dome (SED) identified with a dotted line and germinal center (GC) identified with solid line and white arrow. Scale bars, 100μm.

We next analyzed B cell subset–specific interactions. We filtered the information to interactions that involved genes that were statistically associated with B cell subsets in the CITE-seq data (Fig. 3A). We also split the types of interactions to focus on those we considered to have functional relevance distinct from cell migration, cell retention or antigen presentation (data file S3). The comprehensive diagram of interactions of the five B cell subsets that are enriched in SED in human GALT are represented in Fig. 4A and listed with interactions strengths in data file S3. In general B cell subsets had contrasting interaction profiles. For example, the interaction profile of naïve B cells was dominated by interactions of LTA with TNF receptors that are associated with lymphoid tissue homeostasis and negative regulation of inflammation (33,34). An interaction between *NOTCH2* expressed by MZB and delta ligand like 1 (*DLL1*) expressed by mesenchymal cells was observed, consistent with the predicted potential of MZB to develop from precursors in GALT under the NOTCH2–DLL1 signaling pathway (12, 28, 35).

The most interactive B cells in Fig. 3D and E were the aNAV and DN2 subsets, of which the DN2 were relatively more abundant and localized to the SED. We therefore focused again on the DN2 interactome. Interactions specific to DN2 cells included *TNFRSF13B* with *TNFSF13B* which is a known pathway supporting T cell-independent IgA class switching in the SED region (6, 36). Other interactions of DN2 B cells involve B cell expression of *CD86, GRN, EBI3* and *SIGLEC10* that encode immunomodulatory proteins (37–41). Imputed expression of these genes can be localized mostly to the SED and GC in the CosMx data (Fig. 4B). The strongest imputed interactions of DN2 B cells that had not been identified before were validated as described below. Reagents were not available to enable validation of expression or interactions of *SIGLEC10*.

Interactions of B cells expressing subset marker genes relating to cell migration, retention and antigen presentation were analyzed (fig. S5 and data file S3). DN2 cells were enriched in interactions involving *HLA, B2M* and *CD74*, consistent with their expression of *CD86* and a potential role in antigen presentation (42, 43). The interaction of *CCR7* with *CCL19/21*, that mediates lymphocyte recirculation between lymphoid tissues and blood, was identified in MZB, memory B cells and IgM-only B cells although not in DN2, suggesting a difference in systemic circulation (44). Memory B cells had prominent ITGB1-associated interactions (fig. S5). Thus, B cell subset identification using subset-specific markers reveals their involvement in receptor-ligand interactions contributing to local immune function and regulation in GALT.

### DN2 cells express CD86, GRN, and EBI3 genes and proteins

By analysis of imputed data, CD86, GRN, and EBI3 have been implicated in cellular interactions involving DN2 B cells in the SED of GALT. To validate their specific expression, we iteratively investigated their specific expression on a gene and protein level using mRNA-targeting probes incorporated into IMC imaging, and by immunofluorescence (IF) and confocal microscopy (CM) respectively.

**Fig 5:**
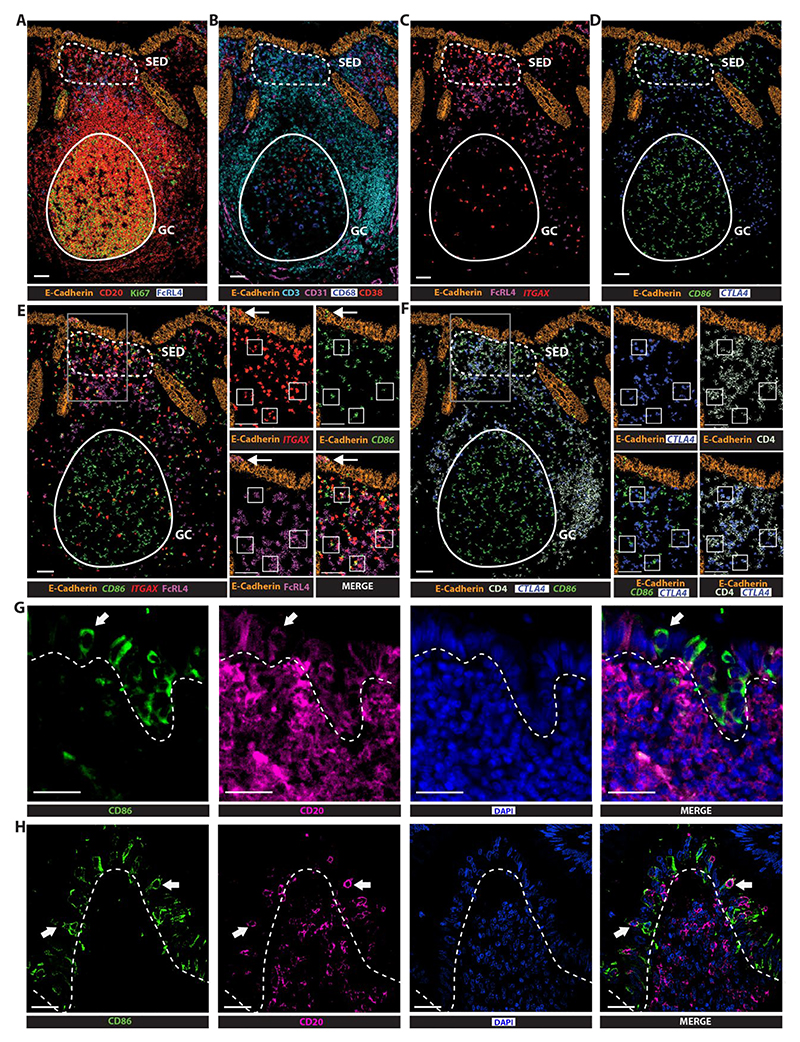
Validation of CD86 gene and protein expression in the SED of GALT. (**A** and **B**) Imaging mass cytometry (IMC) of GALT showing marker features revealing microanatomy, including the relative positions of the epithelium (E-Cadherin), B cells (CD20), FcRL4 (B cells that tend to be close to epithelium including DN2 cells), T cells (CD3), and dividing cells (Ki67) that are enriched in the GC. The sub-epithelial dome (SED) region is identified with a dotted line and the germinal center (GC) with a solid line. (**C**) Location of key markers FcRL4 and *ITGAX* that have enriched expression in DN2 B cells. (**D**) Spatial localization of gene expression for *CD86* and its ligand *CTLA4* are shown relative to the GC and SED. (**E**) IMC of *CD86* expression by B cells with features of DN2 cells in the SED. Arrows in inserts identify an example of an intraepithelial DN2 B cell expressing *CD86*. (**F**) IMC of *CTLA4* expression by CD4 T cells in the immediate microenvironments of *CD86*-expressing B cells illustrating their close proximity. Boxes in inserts in (E) and (F) highlight individual cells that are adjacent and potentially interactive. Data are representative of three biological replicates. (**G** and **H**) Representative (G) immunofluorescence and (H) confocal microscopy showing coexpression of CD86 protein by B cells in the FAE of GALT (arrows). Dotted line indicates the boundary between the epithelium and the underlying tissue. Scale bars, [(A) to (F)] 50 μm and [(G) and (H)] 30 μm.

To validate expression and interactions of CD86, initially an IMC panel including mRNA-targeting probes for *CD86* and *CTLA4* was applied to FFPE sections of human appendix (data file S4). The structural features of human GALT including the distribution of E-cadherin, CD20, Ki67, FcRL4, CD3, CD31, and CD68 and CD38, were initially used to identify microanatomy such as the SED and GC (Fig. 5, A and B). DN2 cells were then identified by their coexpression of FcRL4 and CD11c (encoded by *ITGAX*) (Fig. 5C) and the relative expression of *CD86* and *CTLA4* was visualized (Fig. 5D). *CD86* signal was observed in DN2 cells expressing *ITGAX* and FcRL4, including cells located in epithelium (Fig. 5E). CD4 T cells expressing *CTLA4* were observed in the SED in close proximity to *CD86* mRNA (Fig. 5F). To determine whether CD86 protein was expressed by B cells in the SED and in the epithelium as predicted by gene expression, FFPE sections of appendix were stained with antibodies to CD86 and CD20 (table S2). Coexpression was observed in the intraepithelial and SED compartments by IF (Fig. 5G). Given that the expression of CD86 by M cells that were in close proximity to B cells (5), CM was used to confirm coexpression of CD86 and CD20 (Fig. 5H).

**Fig 6:**
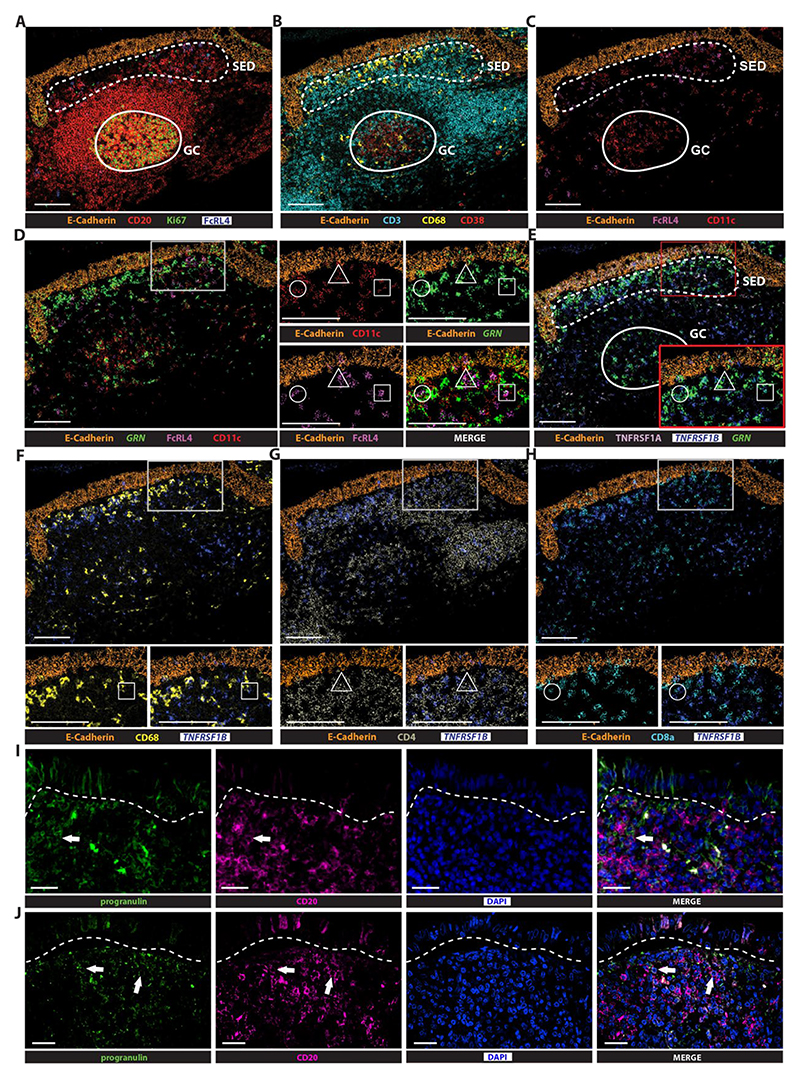
Validation of GRN gene and protein expression in the SED of GALT. (**A** and **B**) Imaging mass cytometry revealing features GALT microanatomy including the relative positions of the epithelium (E-Cadherin), B cells (CD20), FcRL4 (B cells that tend to be close to epithelium including DN2 cells), T cells (CD3), and dividing cells (Ki67) that are enriched in the GC. The sub-epithelial dome (SED) region is identified with a dotted line and the germinal center (GC) with a solid line. (**C**) Location of key markers FcRL4 and CD11c that have enriched expression in DN2 B cells. (**D**) Expression of *GRN* by B cells expressing FcRL4 and CD11c, indicative of DN2 B cells. (**E**) Identification of *GRN* expression alongside expression of binding partners *TNFRSF1A* and *TNFRSF1B*. Compare staining in shapes in inserts in (D) and (E) that aid identification of *GRN* association with cells expressing identifiers of DN2 B cells. (**F**) Expression of *TNFRSF1B* by CD68-expressing myeloid cells in the SED. Shapes highlight locations of individual cells coexpressing indicated markers. (**G**) Expression of *TNFRSF1B* by CD4-expressing cells in the SED. (**H**) Expression of *TNFRSF1B* by CD8 T cells. (**I** and **J**) Representative (I) immunofluorescence and (J) confocal microscopy to identify expression of progranulin protein by B cells in the SED. Arrows in (I) and (J) identify individual cells expressing CD20 and progranulin. Data are representative of three biological replicates. Scale bars: [(A) to (H)] 100 μm and [(I) and (J)] 30 μm.

To validate expression and interactions of *GRN*, initially an IMC panel including probes to detect mRNA encoding *GRN, TNFRSF1A* and *TNFRSF1B* was applied to FFPE sections of human appendix (data file S4). The structural features of human GALT, including the distribution of E-cadherin, CD20, Ki67, FcRL4, CD3, CD68 and CD38, were initially used to identify the SED and GC (Fig. 6, A and B). DN2 cells were identified by coexpression of CD11c and FcRL4 (Fig. 6C). Expression of *GRN* by DN2 cells was confirmed in the SED (Fig. 6D), though most *GRN* was not expressed by B cells. Expression of imputed targets of *GRN, TNFRSF1A* and *TNFRSF1B*, was observed in the SED (Fig. 6E). Cells from within Fig. 6E were further observed to confirm the expression of *TNFRSF1B* by CD68, CD4 and CD8 expressing cells in the same regions of the SED (Fig.6, F to H), where DN2 B cells expressed *GRN*. Expression of the progranulin precursor, that is encoded by the *GRN* gene, by B cells in the FAE and SED was confirmed by IF (Fig. 6I) and CM (Fig. 6J).

**Fig 7:**
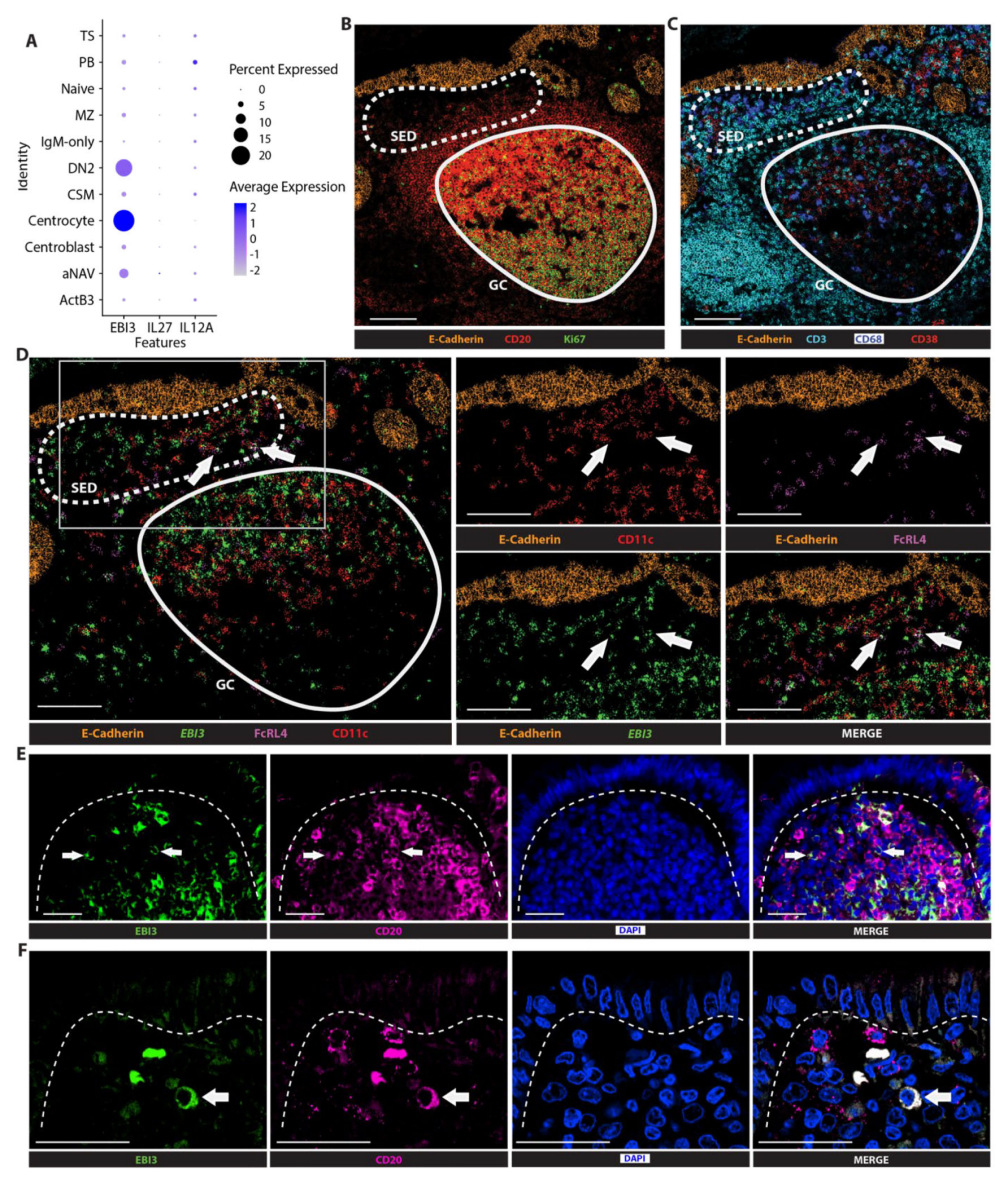
Validation of EBI3 gene and protein expression in the SED of GALT. (**A**) Analysis of of gene expression in CITE-seq data illustrating that *EBI3* is expressed by DN2 cells and centrocytes, but in neither case is expression of *IL27* or *IL12A* observed. (**B** and **C**) Imaging mass cytometry revealing features GALT microanatomy including the relative positions of the epithelium (E-Cadherin), B cells (CD20), T cells (CD3), and dividing cells (Ki67) that are enriched in the germinal center (GC). The sub-epithelial dome (SED) region is identified with a dotted line and the GC with a solid line. (**D**) Selective expression of *EBI3* in the SED and GC alongside FcRL4 and CD11c that are enriched in DN2 cells (**E** and **F**) Representative (E) immunofluorescence and (F) confocal microscopy of EBI3 protein expression by B cells in the SED of GALT. Dashed lines represent the boundary between the epithelium and the underlying tissue. Arrows indicate B cells expressing EBI3. Data are representative of three biological replicates. Scale bars, [(B) to (D)] 100 μm and [(E) and (F)] 30 μm.

EBI3 is a subunit of heterodimeric cytokines IL-27 (with the p28 subunit) and IL-35 (with the p35 subunit) (45). It was identified as an interacting partner because adjacent cells expressed the IL27 receptor component *IL27RA* and the signaling protein *IL6ST* that are known to be expressed abundantly by immune cells (45). We asked whether the p28 subunit of IL27 or the p35 subunit of IL35, encoded by *IL27* and *IL12A* genes respectively, were expressed significantly by DN2 B cells by examining the CITE-seq data and they were not (Fig 7A and data file S1). We therefore validated the expression of *EBI3* using an IMC panel including an RNAscope probe for *EBI3*.

The structure of GALT, including the SED and GC, was annotated by visualizing the distribution of E-cadherin, CD20, Ki67, CD3, CD68 and CD38 (Fig. 7B and C). Expression of *EBI3* gene by DN2 B cells, identified by coexpression of CD11c and FCRL4, was confirmed (Fig. 7D). Expression in the light zone of the GC was also observed consistent with gene expression information in Fig. 7A. Expression of EBI3 protein by B cells in SED was confirmed by IF (Fig. 7E) and CM (Fig. 7F). Thus, we validate the expression of CD86, GRN, and EBI3 by DN2 B cells, revealing specific immunomodulatory interactions with the potential to dampen inflammatory responses in the SED.

### B cells in GALT maintain homeostasis and are dysregulated in ulcerative colitis

The appendix has been implicated in ulcerative colitis (UC) in several contrasting ways including potential protection from UC by early life appendectomy, presence of red patches detetected endoscopically around the appendiceal orifice in UC, even when disease is distal, and also a GALT-associated response to biological therapy (15–18). We applied IMC to investigate cellular interactions in four appendix samples from patients with severe UC who had surgical right hemicolectomy as part of disease management (data files S4 and table S3). Following data acquisition by IMC, imaged cells were segmented, quality controlled (fig. S6A), and cell lineages were identified using a semi-supervised machine learning approach from manually gated data (fig. S6B).

**Fig 8:**
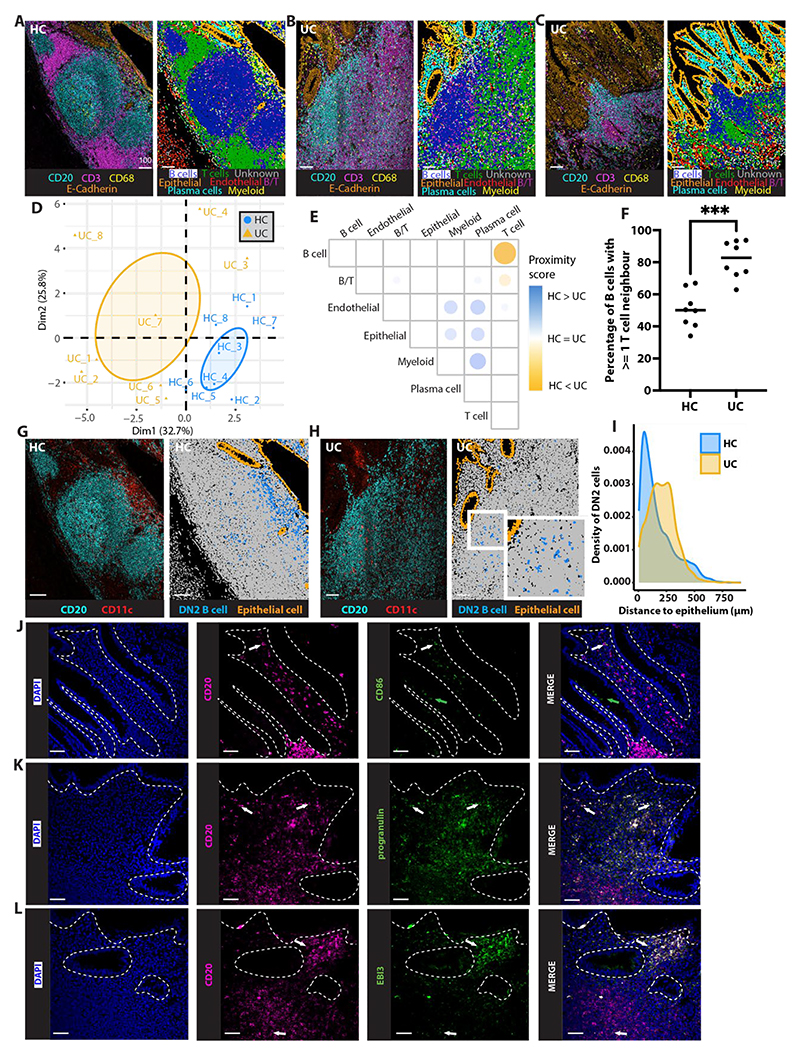
Analysis of B cell subsets in Ulcerative Colitis. (**A**) Imaging mass cytometry (IMC) of healthy appendix (representative of *n* = 4) showing (left) lineage markers and (right) computational classification of cells from the same region of interest (ROI). (**B** and **C**) IMC staining two different UC appendix samples (representative of *n* = 4) showing (left) lineage markers and (right) computational classification of cells from the same ROI in each. (**D**) PCA plot constructed on scaled number of proximities between lineages within each ROI. (**E**) Proximity score between lineages based on interaction score testing between lineages in health and UC. (**F**) Dot plot of percentage of B cells with at least one T cell neighbor in HC and UC (*n* = 4 per group with two technical replicates each). Data were analyzed by two-tailed *t* test). (**G**) IMC of healthy appendix showing (left) DN2 B cell markers and (right) visualization of computationally-derived DN2 B cells. Epithelial cells shown in gray for reference. (**H**) IMC of UC appendix samples showing (left) key DN2 B cell markers and (right) visualization of computationally-derived DN2 B cells. Epithelial cells shown in gray for reference. Insert shows DN2 B cells scattered through the follicle in UC rather than subepithelial clustering. (**I**) Density plot showing distribution of DN2 B cells from HC and UC samples as shown in D, based on minimum distance from an epithelial cell, split by HC and UC. (**J** to **L**) Immunofluorescence showing distribution of CD86, progranulin, and EBI3 expression respectively in UC appendix. Representative of *n* = 3. Dotted lines indicate the boundary between epithelium and the underlying tissue. Arrows identify CD20-expressing B cells coexpressing CD86, progranulin, and EBI3, respectively. Scale bars, [(A) to (C) and (G) and (H)] 100 μm and [(J) to (L)] 50 mm.

The distribution of key lineage markers and markers indicating B cell zonation were visualized alongside computationally derived lineages to ensure that they were consistent for healthy control (HC) appendix (Fig. 8A) and examples of appendix from patients with UC (Fig. 8, B and C). Neighborhood graphs were constructed per image, and the proportion of lineage neighbors per region of interest (ROI) was determined. A principal component analysis (PCA) constructed on the lineage-based edge types showed that the HC samples were similar in their neighborhood composition, whereas the UC samples exhibited much greater heterogeneity and were distinct from HC samples (Fig. 8D). Proximity testing performed to investigate these compositional differences, identified a greater tendency for B cells and T cells to be neighbors in UC compared to HC. This is consistent with the loss of B cell and T cell zonation (Fig. 8E). Comparison of the number of B cells with at least one T cell neighbor validated that B and T cells had a greater tendency to be intermixed rather than zonal in UC (Fig. 8F).

B cell subsets were identified based on clustering the labelled B cells in HC (fig. S6, C to E) and UC (fig. S6, F to H) samples, separately. When DN2 cells were visualized on the images (Fig. 8, G and H), they clustered closer to the epithelium in HC than in UC, confirmed by a difference in distribution of DN2 when based on their minimum distance to an epithelial cell (Fig. 8I). We determined the expression of CD86, EBI3 and progranulin by B cells in UC mucosa. Low B cell expression of CD86 was observed in UC samples, despite detection of some non-B cells in epithelium expressing CD86 (Fig. 8J). By contrast, progranulin (Fig. 8K) and EBI3 (Fig. 8L) were both expressed abundantly by B cells located close to the epithelium in UC.

Thus, cell lineages and B cell subsets had altered distributions, and different profiles of cellular interactions in UC compared to HC. Modified expression of CD86, EBI3 and progranulin by B cells in UC are likely to contribute to altered local immune regulation. In HC, DN2 B cells had the highest number of cell-cell interactions of all GALT B cell subsets and were more strongly localized to the SED compared to UC. Thus, B cells in GALT contribute to the preservation of a microenvironment that maintains immune homeostasis and their dysregulation may contribute to inflammatory bowel disease.

## Discussion

The FAE that overlies the lymphoid tissue in GALT actively samples microbes into lymphoid tissues (1). We sought to understand the interactions of B cells on this antigenic frontline that contribute to the maintenance of tissue homeostasis in the presence of innate ligands. We identified that the most interactive B cells in human GALT were aNAV and DN2 B cells. Of these cell subtypes, DN2 B cells were most abundant in and localized to the SED region, which is where antigens, including bacteria, are actively sampled from the gut lumen by the immune system (2–4). We therefore focused on the DN2 B cell subset and observed that their dominant interactions were immunomodulatory in nature.

There is already precedent for some imputed interactions from studies of in vivo mouse models and human cells in vitro (6, 36, 48, 49). For example, here we identified that DN2 B cell expression of *TNFRSF13B*, which encodes transmembrane activator and CAML interactor (TACI), interacted with myeloid cell-expressed *TNFSF13B*, encoding B-cell activating factor (BAFF). Such interactions of TACI have been linked with T cell–independent activation of B cells. Ligation of TACI can substitute for ligation of CD40 in the up-regulation of activation induced deaminase (AID) supporting class switch recombination in the SED where DN2 B cells tend to locate (6, 36, 48, 49).

The interaction of CD86^+^ DN2 B cells with CTLA4^+^ CD4 T cells was observed in the SED and the FAE. Expression of CD86 by B cells in this microenvironment has been observed previously (50). It is likely that this is an immunosuppressive interaction that down-regulates T cell activity, however CD86 has the potential to be proinflammatory when interacting with CD28 (40, 42, 43). In addition, DN2 B cells strongly expressed genes encoding for markers that are associated with antigen presentation, including *HLA-B* and *B2M*. This suggests that the expression of CD86 by B cells is tightly associated with the antigenic frontline and the GC, which could be critical in deciding the type of T cell response in this microenvironment (40, 42, 43). Furthermore, interactions between DN2 B cells expressing GRN, and TNF receptors expressed by myeloid cells and CD4 and CD8 T cells were imputed and validated, though it should be noted that we observed both B cells and myeloid cells expressing GRN in the SED. It has been proposed that granulin can inhibit the interaction between TNF and its receptors and thus could be an important negative regulator of proinflammatory TNF activity in the SED (37, 38, 51).

*EBI3* was identified in the list of interactions by its association with IL27RA, expressed by CD4 and CD8 T cells, and IL6ST expressed by CD4 T cells, endothelial cells, mesenchymal, and myeloid cells. *EBI3* encodes the B subunit of immunomodulatory cytokines IL27 and IL35 (39, 45). However, only *EBI3* was expressed by DN2 cells, but not the p28 or p35 subunits of IL27 and IL35, respectively. The interactions with IL27RA and IL6ST were likely captured because both IL27RA and IL6ST are reported to be widely expressed by lymphocytes (45). Expression of EBI3 alone has been reported to have immunoregulatory functions, due to its ability to interfere with the binding of various cytokines to receptors, including the binding of IL6, thereby forming a “cytokine sink” to prevent inflammatory responses (39, 46, 47).

In addition to the immunomodulatory interactions observed in this study, we identified FcRL4 expression in the SED and FAE. FcRL4 is an inhibitory receptor that dampens the consequences of BCR ligation and is expressed by B cells in proximity to the mucosal epithelium (52–55). Enriched expression of FcRL4 by DN2 B cells is consistent with the immunosuppressive profile of B cells in the SED and further highlights the importance of a tightly regulated immune responses within this antigen-rich microenvironment. We identified that most B cells in GALT expressed *IL16*. IL16 is a chemoattractant for CD4-expressing cells. It is possible that B cell–derived IL16 could direct the migration of CD4-expressing cells in tissue microenvironment, including the SED, and contribute to intestinal homeostasis and inflammation (56, 57).

Our manuscript provides a resource of B cell subset interactions with other cell lineages. We identified that the *NOTCH2* gene is a marker of only MZB cells and that the protein has potential to interact with mesenchymal DLL1 in GALT. These observations support that MZB development and maturation are derived from precursors dependent on NOTCH2–DLL1 in the GALT microenvironment (12, 28, 35). Furthermore, interactions between naïve B cell LTA and TNF receptors are associated with the maintenance of lymphoid architecture and structure, including GALT and the spleen (33, 34).

We identified parameters associated with cell migration and antigen presentation involved in the orchestration of tissue architecture. For example, the interaction of CCL21/CCL19 with CCR7, associated with lymphocyte recirculation, was identified for MZB, class switched memory, and IgM-only B cells but not DN2 B cells (44). This suggests that DN2 B cells are not migratory, consistent with their low frequencies in healthy blood (24, 44). The interaction between B cell CCR6 and mesenchymal CCL20 was observed for MZB cells and IgM-only B cells. This interaction has been implicated in the cellular localization to the SED in mice (58, 59) and could be associated with the SED and perifollicular distribution of MZB cells and IgM-only B cells, but not DN2 B cells.

The appendix has been implicated in the disease process of UC, whereby reddening around the appendiceal orifice in UC, termed “periappendiceal red patches”, can occur even when colitis is distal (17), and an appendectomy in early life can be protective (15, 16, 60). The importance of GALT in UC has been demonstrated by the impact of vedolizumab, which blocks a4b7-MAdCAM1–mediated homing into gut tissues, and the ensuing GALT structure observed in clinical response (18). Here, we observe that normal cellular interactions in GALT are disrupted in severe UC. In addition to the lack of B cell and T cell zonation, which increases the potential for B cell–T cell interaction, we observed that DN2 B cells that can express TACI and BCMA that receive immunomodulatory signals in health lose their subepithelial location and are thus functionally displaced. In addition, B cell expression of CD86 is lost and progranulin and EBI3 become abundantly secreted by B cells. It is unclear if B cell–derived EBI3 and progranulin contribute to the inflammatory response or represent a compensatory response to curb inflammation. These observations highlight the importance of considering B cells as immunoregulators when working to understand lymphoid tissue function and mechanisms of inflammation.

This study has limitations including our analysis of only B cells and adjacent interacting partners. It is likely that the immunomodulatory capacity of B cells extends beyond the interactions observed here. Our analysis of UC was restricted to severe disease. UC is a chronic relapsing disease with various degrees of severity in individuals across a lifetime and the features of severe UC we described here will not be observed in all patients. However, we identify potential roles for B cells that could contribute to the diseased mucosa besides antibody production.

We have generated a spatial and functional atlas of local B cell interactions in human GALT by integrating multimodal data, including CITE-seq and spatial transcriptomics. The immunoregulatory features of DN2 B cells that preferentially locate in the SED potentially support the maintenance of lymphoid tissue homeostasis in a microenvironment that is rich in pathogen-associated molecular patterns, thus allowing the chronic propagation of B cell responses against the bacterial surface without inducing inflammation (6, 7, 48).

## Materials and Methods

### Study design

This study sought to understand the spatial localization and potential interactions of cells in human gut-associated lymphoid tissues (GALT) to determine cell functionality and changes associated with the inflammatory bowel disease, ulcerative colitis (UC). Single-cell transcriptomics was used to profile immune cells in GALT and investigate cell-cell communication, including coupling with single-cell RNA sequencing (scRNA-seq), which allowed for increased depth of information with a spatial context. Imaging mass cytometry (IMC) incorporating RNAscope mRNA probes and fluorescence-based methods were used to validate interactions. IMC and immunofluorescence (IF) methods were used to investigate interactions in UC mucosa to localize cell populations between conditions.

### Human tissue samples

Please see table S3 for details of human samples used for this study. Human appendix cells for CITE-seq analysis were obtained from three deceased adult transplant organ donors at the Department of Surgery, University of Cambridge and NIHR Cambridge Biomedical Research Centre with research ethics committee (REC) approval and informed consent from the donor family (reference 15/EE/0152, East of England Cambridge South Research Ethics Committee). Eight of the twelve FFPE samples of normal appendix were collected from surgical theatres between 1983 and 1996 as material from anonymous donors that would otherwise have been discarded. Four further samples were provided anonymised from pathology archives of Guy’s and St Thomas’ Hospitals. All were from tissue donors who had undergone appendicectomy or right hemicolectomy. Seven of these histologically normal FFPE appendix samples were used for CosMx analysis. Four further samples were used for validation. Four samples from the same collection, were used for comparison with four samples of UC appendix. No information is available relating to the 12 normal tissue donors. Studies of human cells and tissues for this project was approved by London, Camberwell St Giles Research Ethics Committee (study 11/LO/1274 Immunology of the intestine; features associated with autoimmunity).

### Sample processing

Appendix samples were collected from three deceased transplant organ donors and were processed as described previously (26, 61). All tissue preparation and lymphocyte isolation procedures were performed with Roswell Park Memorial Institute (RPMI) 1640 medium (GIBCO, 61870-010) containing heat-inactivated 10% fetal bovine serum (FBS) (Sigma Aldrich, F7524), l-glutamine (2 mM) (Sigma-Aldrich, G7513), penicillin (100 IU/ml), and streptomycin (100 mg/ml) (RPMI-P/S) (Merck Life Science UK, p4333) unless stated otherwise. Briefly, appendix was cut into 1- to 2-mm pieces and incubated at 37°C for 30 min in medium with collagenase IV (1 mg/ml;Sigma-Aldrich) and deoxyribonuclease (DNase) I (1 mg/ml; Roche). Lightly digested appendix was then washed with medium and resuspended for cryopreservation. Cells were cryopreserved in freezing medium of FBS supplemented with 10% dimethyl sulfoxide (DMSO) (Sigma Aldrich, D2650) in aliquots of 1 × 10^7^ cells.

### Cell sorting, CITE-seq staining and scRNA-seq library preparation

Cryopreserved samples were thawed in a 37°C-water bath and washed in RPMI 1640 containing 10% FBS. After cell counting, 3 x 10^6^ cells per sample were resuspended in Dulbecco’s phosphate-buffered saline (DPBS) (Gibco, 14190-094) with 1% bovine serum albumin (BSA) (Merck Life Science UK, A1595) that we refer to as FACS buffer, then transferred to Eppendorf LoBind microcentrifuge tubes, and washed with FACS buffer. Cells were blocked on ice with Human TruStain FcX (BioLegend, 422302), diluted 1:10 in FACS buffer for 10 minutes on ice and then stained for 30 minutes on ice with oligonucleotide-tagged antibodies (table S1) and PE-dazzle anti-CD19 antibody (BioLegend, 363031) and washed twice in FACS buffer before sorting CD19 expressing cells using a BD FACSAria IIu SORP (BD Biosciences) cell sorter equipped with BD FACSDiva software (v8.0.1), with a 100 µm nozzle and at low-pressure (20 PSI) to avoid cellular stress and increase cell viability. Sorted CD19^+^ cell populations from the three donors were loaded onto a 10X Genomics Chromium controller, and the libraries [5′ gene expression, V(D)J, and antibody derived tags (ADT)] were prepared according to the manufacturer’s guidelines. Sequencing data were generated using Illumina NextSeq 2000 platform (28-10-10-90 sequencing configuration). Transcript alignment and generation of feature barcode matrices for downstream analysis were performed using the 10X Genomics Cell Ranger workflow.

### Analysis of CITE-seq data

Gene expression and surface antibody (ADT) levels for the CD19^+^ cells were loaded into R using the Seurat package (version 5.0.3) (62). Quality control removed cells with high mitochondrial read percentage, low ribosomal read percentage, high or low gene counts and read counts, with thresholds determined per sample by exponentiating the median plus (for maximum values) or minus (for minimum values) three times the median absolute deviation of the log-transformed values; providing a robust upper cutoff for identifying outlier values. Cells with no expression of B cell lineage markers *CD79A, CD79B, CD19* and *MS4A1* were also excluded.

Expression data was normalized for each sample (SCTransform for the RNA assay, centered log ratio for the ADT assay). For the RNA assay, the top 3000 genes with the most variation, excluding *IGHV* genes, were used for principal component analysis (PCA) construction. A PCA was constructed for each of the ADT assays, and a weighted-nearest neighbors method (63) was used to integrate samples and produce PCA and uniform manifold approximation and projection (UMAP) dimensionality reductions using both assays combined.

Cells were then clustered based on the combined PCA and clusters were manually assigned a label based on the expression of known B cell subset genes and surface markers. Clustering was performed with the FindClusters function, with resolution set to 2.0 and using smart local moving algorithm. Where necessary, clusters were further subclustered (using FindSubclusters with resolution set at 1.0) to provide better resolution. Gene markers for each B cell subset were calculated using the Seurat FindMarkers function with default parameters (Wilcoxon rank sum test with correction for multiple comparisons).

### CosMx sample processing

Sections from seven FFPE blocks of normal human appendix cut at 5-µm thickness were deparaffinized in xylene (XYL005, Solmedia) and two washes in 100% ethanol (Sigma-Aldrich, 32221) for 2 min each. All of the following steps were then carried out using kit reagents and protocols supplied by Bruker (previously NanoString) unless otherwise stated. Slides were subjected to target retrieval (100°C for 15 min) and proteinase-K digestion [3 µg/ml in DPBS, 40°C for 30 min) according to manusfacturer’s instructions for appendix (Bruker Supplied Reagents, CosMx FFPE Slide Preparation Kit (RNA), 121500006]. For image registration, the concentration of fiducials was 0.0005% in 2X SSCT (RT for 5 min). Slides were then washed once in DPBS to remove excess fiducials, and fixed with 10% neutral buffered formalin (HT501128, Sigma-Aldrich) for 1 min at room temperature. Fixation was stopped with two washes of Tris-glycine buffer, consisting of 0.1 M glycine (Sigma-Aldrich, G8898) and 0.1 M Tris-base (Sigma-Aldrich, T1503), followed by one wash with DPBS for 5 min. Slides were blocked with 100 mM NHS-acetate (Thermo Scientific, 26777) in CosMx NHS-acetate buffer (Bruker Spatial Biology) for 15 min at RT, followed by two washes with 2X SSC for 5 min each. The NanoString RNA probe set Human Universal Cell Characterization Panel 1000 plex (RNA) was prepared according to manusfacturer’s instructions and applied to the slides and incubated at 37°C in a hybridization oven overnight (18 hours). The following day, the slides were washed twice in 50% formamide (Fisher Scientific, AM9342) with 50% 4X SSC at 37°C for 25 min per wash, then rinsed twice in 2X SSC for 2 min at RT. For nuclear staining, slides were incubated with 4′,6-diamidino-2-phenylindole (DAPI) at room temperature for 15 min, protected from light. Cell segmentation was performed using Human Universal Cell Segmentation Kit (RNA) consisting of CD298/B2M, PanCK and CD45, with a 1-hour incubation at room temperature and protected from light, followed by three washes in PBS. Flow cells (Bruker Spatial Biology) were loaded onto slides, and the slides were then loaded onto the CosMx SMI instrument. Data were acquired using the pre-bleaching profile (Configuration A) and cell segmentation profile (Configuration A) (64). Data were acquired as *z*-stacks at 0.8-μm intervals (65). The detection of transcript dispersion and their removal in specific *Z*-slices was performed using the R package NoButter (66). Dispersion was observed in *Z*-slices higher than *Z*8, so all transcripts present in these slices were removed. The resulting cell gene expression and spatial coordinates were used for downstream analysis.

### Analysis of CosMx cell data

CosMx gene expression and spatial location data were loaded into R using the Seurat package. Quality control was performed on the cells to remove any cell with low (< 20) or high (> 1000) gene reads, and to remove cells with a high proportion of reads (20%) corresponding to the negative control probes. The RNA assays for each of the CosMx samples were normalized individually using the SCTransform function, and PCA and UMAP dimensionality reductions were calculated for each sample. Distinct clusters exhibiting epithelial cell markers (*KRT18, CD9, EPCAM*, and *CDH1*) were identified and these were classified as epithelium. Other clusters exhibited mixed cell lineage markers and thus for the remaining cells, a label transfer method was used. Previously published, prelabelled data taken from cells in the human intestinal tract (19) was used and filtered to only include cells corresponding to adult appendix to best match the CosMx samples. Epithelial and neuronal cells were removed resulting in a reference dataset containing cells labelled as B cells, T cells, endothelial cells, myeloid cells, and plasma cells. The data were normalized using SCTransform and a PCA was computed for this reference dataset using the top 3000 most variable genes. The FindTransferAnchors function in Seurat (20) was used to find anchors between cells in the CosMx and cells in the reference dataset based on transcriptomic similarity in the 480 genes present in both datasets. The TransferData function was then used to assign a lineage classification based on the classification of anchored reference cells.

Cell-cell proximity was determined using the imcRtools package (67), involving Delaunay triangulation with a cutoff of 125 microns between cell centers to prevent proximities occurring across large distances. Edges were assigned between neighboring cells to construct a network, and each cell was given a neighborhood lineage similarity score calculated as the proportion of the cell’s neighboring cells that share the same lineage as the cell. Proximity testing was performed using the testInteractions function in imcRtools. Briefly, proximities between cells were calculated for each sample, and a bootstrapping approach was used whereby lineage classifications were shuffled and recorded 1000 times. Observed proximity numbers for each combination of lineages were then compared against the shuffled repetitions to give a positive, negative, or neutral proximity inclination per sample. These values were then summed across samples to give a sum of significant proximity.

### Analysis of CosMx B cell data

The CosMx data were filtered to include only cells labelled as B cells, and the anchor-based data transfer approach was repeated, now using the CITE-seq human appendix data as the reference dataset using the 238 genes overlapping between the CosMx genes and the RNA-seq genes used for integration in order to match cells between the datasets based on their transcriptomic similarity. B cell subset labels were transferred, and an RNA assay expression was imputed using this approach, generating the spatial imputed gene expression within the CosMx B cell data of the 3000 genes used for integration in the CITE-seq B cell data. B cell subset proximities were calculated using the same bootstrapping method as described previously.

### Analysis of CosMx ligand-receptor pairs

Ligand-receptor analysis was performed on the CosMx dataset. First, the CellTalkDB database of known interacting source and target cell pairs was downloaded (32) and filtered based on whether the genes involved were present in the CosMx panel or in the imputed B cell RNA assay. Only ligand-receptor pairs with at least one gene in the CosMx panel (‘observed’) and one gene either observed or in the imputed RNA assay (‘imputed’) were retained. Each edge in the previously constructed cell-cell proximity network was converted to a directed network with two edges between neighbors, with one node designated a ‘source’ and the other designated a ‘target’. For each edge, each ligand-receptor pair was given a strength at the edge based on the ligand expression at the source node and the receptor expression at the target node. Total strengths per edge were calculated by summing strengths across all ligand-receptor pairs. Ligand-receptor pairs were deemed to be present between cell types if the interaction strength was > 0 across at least 10% of edges between each cell type pair. For interaction analysis, B cells were divided into their assigned subsets, while cells classified as T cell were reclustered, with clusters identified as CD4^+^ or CD8^+^, and T cells subdivided along these lines into CD4^+^ T cells and CD8^+^ T cells. Mean interaction strength per edge between cell types was calculated, and total interaction strength across edges was visualized spatially. Pathway analysis was performed using Metascape (68), whereby the inputs for each subset were the genes involved in interactions involving that subset.

To identify interactions associated with B cell subset function, individual ligand-receptor pairs were further filtered to only include pairs where the B cell gene was also present in the CITE-seq marker gene for the subset. The strength of these interactions was then visualized across B cell subsets and their interaction partners.

### Mitigation of false positives from ligand-receptor analysis and prioritization of interactions for validation

We considered the following to be key for mitigations of false positives in the data: i) accurate lineage designations at the outset, ii) restricting the analysis to interactions involving at least 10% of edges between each cell type pair, iii) using a database of interactions that was fully referenced and that could be filtered to interactions involving human cells, iv) giving priority to interactions involving statistically significant marker genes of the B cell subsets, and v) dividing the interactions into those involved in cell migration or maintenance, potential antigen presentation, and immune function, with emphasis on immune function

B cell subset interactions were initially identified by gene expression derived from scRNA-seq data that were projected computationally onto the spatial transcriptomic data. It was therefore important to know if examples that we considered to be functionally relevant because of high interaction strength and association with B cell function in the SED could be identified directly by gene and protein expression. Genes selected by these criteria and by availability of reagents for validation in the sections below were *CD86, GRN*, and *EBI3*.

### Tissue imaging

Immunofluorescence (IF) and confocal microscopy (CM) analysis of FFPE tissues (three biological replicates for each) were carried out as described previously using the Olympus BX51 fluorescence microscope and Nikon AXR inverted confocal microscope with Nikon Spatial Array Confocal respectively. Antibodies are detailed in table S2 (4). IF images were acquired using a x20 objective lens and CM images using a 60× oil-immersion objective with Numerical Aperture 1.4. Confocal images were acquired using 405 nm, 488 nm, and 561 nm laser lines, selected using NIS-Elements dye based experiment presets and channel settings. Images were processed using Fiji.

For IMC, slides were deparaffinised, rehydrated, and subjected to antigen retrieval using VisUCyte antigen retrieval reagent – basic (R&D systems, VCTS021) by pressure cooker. Tissues were then blocked and incubated with the mix of metal-conjugated antibodies specified in the panel overnight at 4 °C and subsequently incubated with the DNA intercalator Iridium Cell-ID Intercalator-Ir (Standard BioTools) before being air-dried as detailed previously (69). Data was acquired as described previously using Hyperion Imaging System (three biological replicates for each) (69). For the IMC-RNAScope combined protocol, expression of genes encoding *IL16, CD86, CTLA4, EBI3, IL27RA, GRN, TNFRSF1A*, and *TNFRSF1B* were detected using RNAscope probes from Bio-Techne as described previously (4). Probes were detected by incorporation of metal-tagged antibodies to digoxigenin, FITC, and biotin into an IMC panel as described before (4) and detailed in data file S4. IMC image data was preprocessed using IMCSegmentationPipeline (70, 71), and analyzed according to further established pipelines (67, 71, 72), as described previously (4).

A lineage classification approach was performed whereby cells were gated based on expression of lineage markers, with cells passing through multiple lineage gates left unlabeled. A population of B cells and T cells was gated based on double expression of CD3 and CD20. A random forest classifier was then trained on the gated cells to apply a label to the unlabeled cells. First, the B cell and T cell population was classified based on a classifier trained on the gated B cells and T cells, assigning cells from the B cell and T cell population into either B cell or T cell classifications. Then, a classifier was trained on the entire labeled population (excluding the B cell and T cells) and used to classify the remaining unlabeled cells. For both classifications, cells with an uncertain classification (defined as a maximum classification probability less than 33% greater than the second highest classification probability) were left unclassified. Proximity edges between cells were calculated based on a distance threshold and the number of edge types was determined for each ROI based on the lineages present on either end of the edge. This data was scaled per ROI and used to construct a PCA of cell proximities. Proximity testing was again performed using the testInteractions function for both health and UC. A proximity score was calculated by subtracting the UC score from the healthy score, giving a metric for determining if cell lineages were in closer proximity in health versus in UC.

The data were then filtered to B cells and reclustered, with the markers used for clustering chosen for their B cell relevance and markers were excluded if they exhibited low signal-to-noise ratio in the healthy B cell data. Health and UC B cells were clustered separately, and B cell subset classifications were assigned based on marker expression, with clusters exhibiting no positive expression left as unknown. For DN2 B cells, the minDistToCells function in imcRTools was used to determine the minimum distance of each DN2 cell to epithelial cells.

### Statistical analyses

For differential gene expression in RNA-seq and CosMx data, Wilcoxon tests with Bonferroni correction for multiple comparisons were used in the Seurat package in R version 4.3.1. All other statistical tests were done in Prism v10. Two-tailed paired *t* tests were performed using parametric tests for comparisons of health and UC in mass cytometry data.

## Supplementary Material

Data File S1

Data File S2

Data File S3

Data File S4

Data File S5

Supplementary Figures

## Data Availability

Samples from deceased donors were transferred from Cambridge University to King’s College London under a materials transfer agreement.. Code for CITE-seq, CosMx and IMC analysis is available at (73). CITEseq data is available on GEO, accession numbers GSM9656504 to GSM9656515. CosMx and IMC data, and code for CITE-seq, CosMx and IMC analysis are available at (74). Tabulated data underlying the figures are provided in data file S5. All data needed to evaluate the conclusions in the paper are present in the main text or the supplementary materials.

## One-sentence summary

B cells in human gut-associated lymphoid tissue support mucosal homeostasis by being highly interactive and immunomodulatory.
