# Supplementary material for: Atlas of human gut-associated lymphoid tissue reveals immunomodulatory interactions of B cells
Source: Sci Immunol. Author manuscript; Available in PMC 2026 Jul 25. (PMC7619268; doi:10.1126/sciimmunol.ady8948)
Supplement: Supplementary Figures [file EMS216124-supplement-Supplementary_Figures.docx]

Supplementary Materials for

**Atlas of human gut-associated lymphoid tissue reveals immunomodulatory interactions of B cells**

Michael J Pitcher, Xiaowen Sun, Chiara Dionisi, Lucia Montorsi, Sherine H Kottoor, Jacqueline HY Siu, Roman Laddach, Rosamond Nuamah, Gavin J Pettigrew, Richard J. Ellis, Cynthia Bishop, Jahangir Sufi, Pawan Dhami, Heli Vaikkinen, Audrey Kelly, Anna Vossenkamper, Deena L. Gibbons, Jo Spencer

Corresponding author: [jo.spencer@kcl.ac.uk](mailto:jo.spencer@kcl.ac.uk)

**The PDF file includes:**

Figs. S1 to S6

Tables S1 to S3

**Other Supplementary Materials for this manuscript include the following:**

Data file 1: Lineage and B cell marker genes

Data file 2: Definitions of B cell subsets

Data file 3: Data relating to interactions.

Data file 4: Imaging mass cytometry antibody panels

Data file 5: Figure data

MDAR Reproducibility Checklist


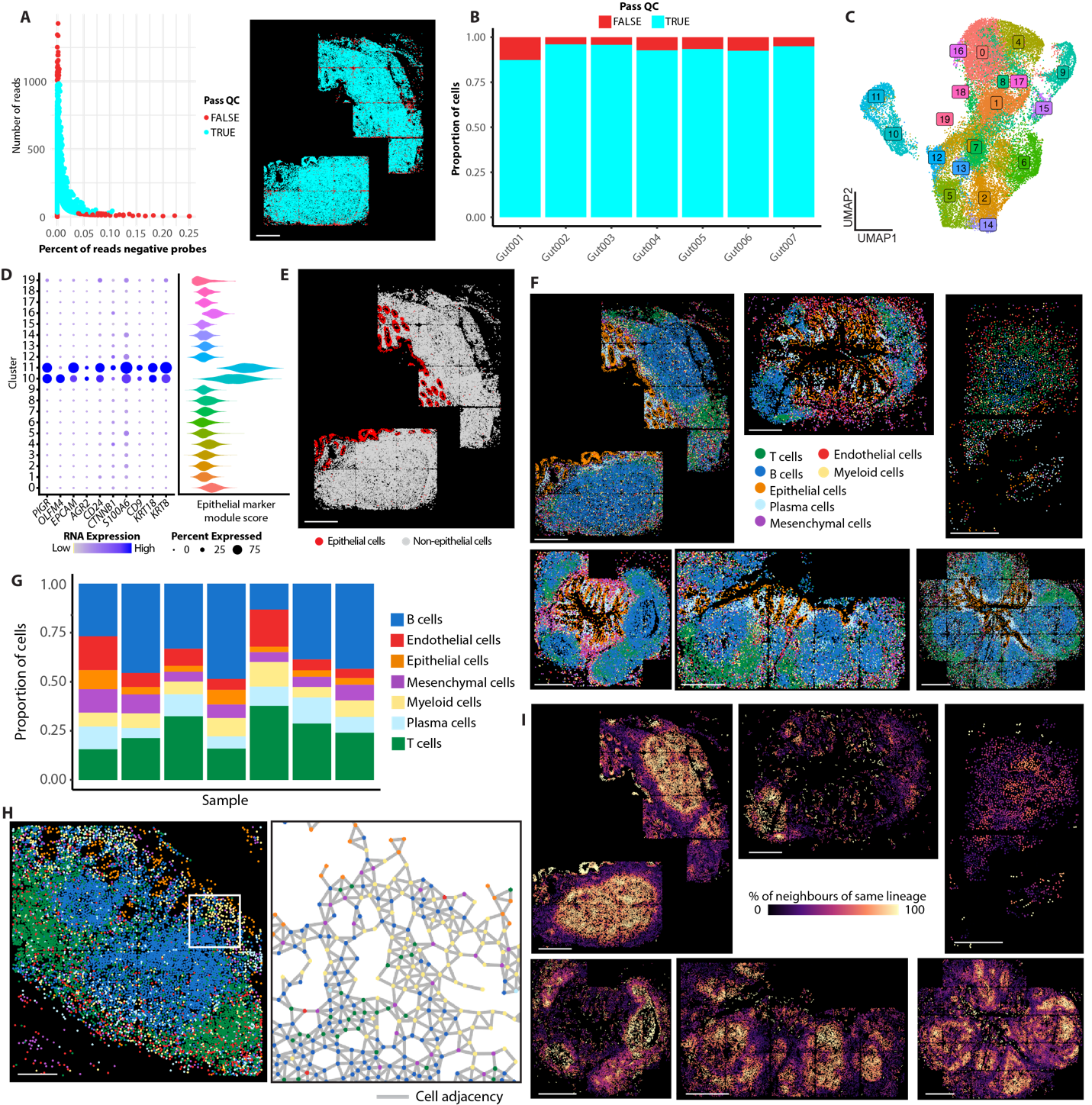


**Fig. S1. Computational analysis of cells in GALT using CosMx**

(A) Quality control (QC) of cells from a single representative sample in CosMx data, based on manual thresholding of reads per cell and percentage of reads belonging to negative probes (left). Resulting classifications of pass/fail QC were visualised back on the images (right). (B) Bar chart showing proportion of cells passing QC for each CosMx sample. (C) UMAP visualization of clusters from the same sample as shown in (A – labelled Gut004 in (B)). (D) Dot plot (left) showing expression of epithelial cell markers in clusters from (C). Genes are top 10 differential expressed genes from cells labelled as ‘epithelial cells’ in reference data used for lineage labelling in Fig. 1. Violin plot (right) showing module score per cluster for all epithelial markers. (E) Spatial plot showing position of cells from clusters 10 and 11 in (D) marked as ‘Epithelial cells’. (F) Spatial plots for cell lineages for other CosMx samples – sample labelled Gut003 is shown in Fig. 1B. (G) Stack bar plot showing the proportion of major cell linages across individual samples using CosMx. (H) Spatial plot demonstrating construction of neighbor graphs by Delaunay triangulation. (I) Spatial plot showing percentage of neighbors of each cell which share the same lineage as the cell for CosMx samples – Gut003 is shown in Fig. 1G. Scale bars: [(A), (E), (F) and (I)] 500μm and (H) 100 μm.

**
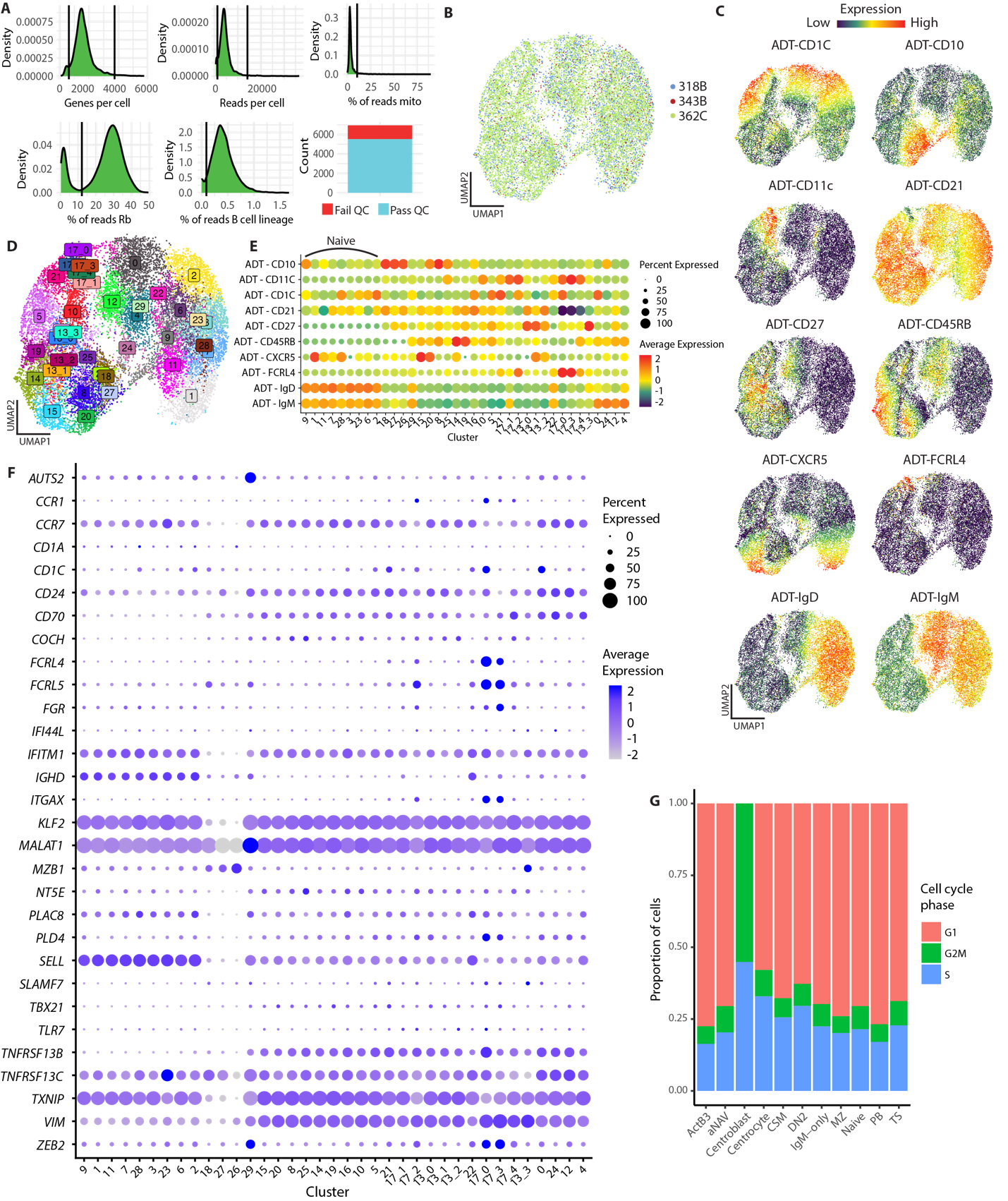
**

**Fig. S2. Computational analysis of B cells in GALT using CITE-seq**

(A) Quality control from a single representative sample, showing automatic thresholding for metrics: genes per cell, reads per cell, percentage of reads per cell classified as mitochondrial, percentage of reads per cell classified as ribosomal, percentage of reads per cell belonging to B cell lineage genes (*CD19, MS4A1, CD79A, CD79B*). (B) UMAP visualization of B cells, showing cells split by donor of origin for confirmation of batch effect integration methods. (C) UMAP visualization of ADT surface antibody markers. (D) UMAP visualization of cells colored by cluster before manual cluster re-labeling. (E) Dotplot showing expression of ADT cell surface markers for each cluster in (D). (F) Dotplot showing expression of RNA markers for each cluster in (D). (G) Bar chart showing proportion of each labeled B cell subset belonging to cell cycle phases G1, G2M and S.


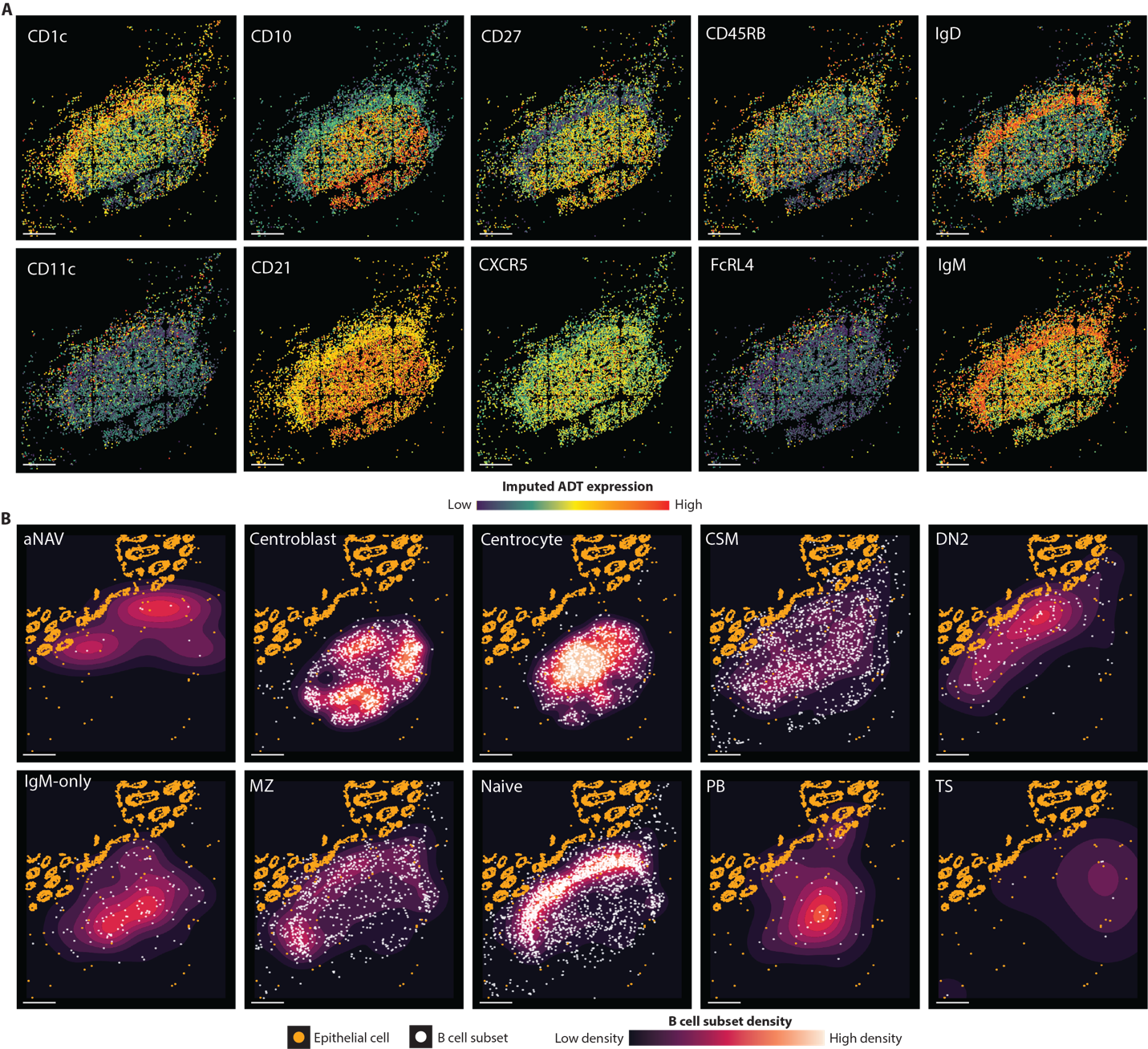


Fig. S3. Validation of imputed B cell subsets in GALT using CosMx

(A) Spatial visualization of imputed cell surface markers across B cell subsets in CosMx data. (B) B cell subset densities across the follicle shown in Fig. 2E. Scale bars: [(A) and (B)] 100μm.

**
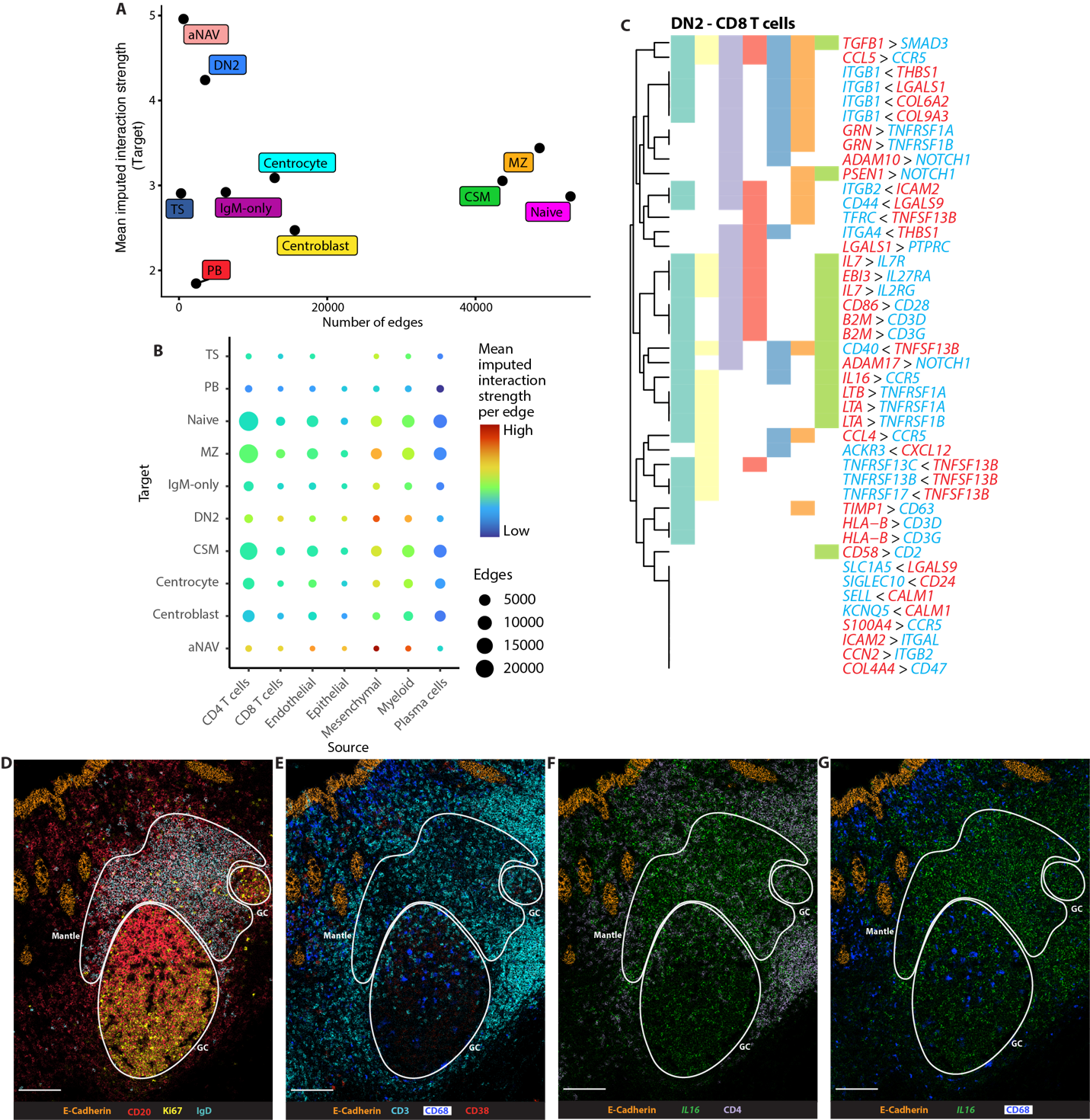
**

**Fig. S4. Imputed interaction networks of B cell subsets and spatial validation of *IL16* gene expression in follicular microenvironments**

(A) Scatter plot showing the number of interaction edges (x-axis) and the mean imputed interaction strength (y-axis) for each B cell subset. Values include interactions where cells act as target (expressing imputed receptor). (B) Dot plot for B cell subset imputed interactions with B cells as source (expressing imputed ligand) and other lineages as the target (expressing observed receptor). (C) Ligand-receptor pairs present on DN2 interactions with CD8 T cells.

(D-G) Representative images generated by IMC and RNAscope of *IL16* transcript expression in follicular architecture with germinal centers (GC) and mantle zones outlined in white. (D) B cell compartment (CD20) and proliferative status (Ki67). (E) Distribution of T cells (CD3), myeloid cells (CD68) and plasma cells (CD38) regions. (F) Spatial distribution of *IL16* transcripts near CD4 T cells across follicular and GC areas. (G) *IL16* transcript regions are spatially adjacent to CD68 myeloid cells within follicular microenvironments. Scale bars: [(D) to (G)] 100 μm.


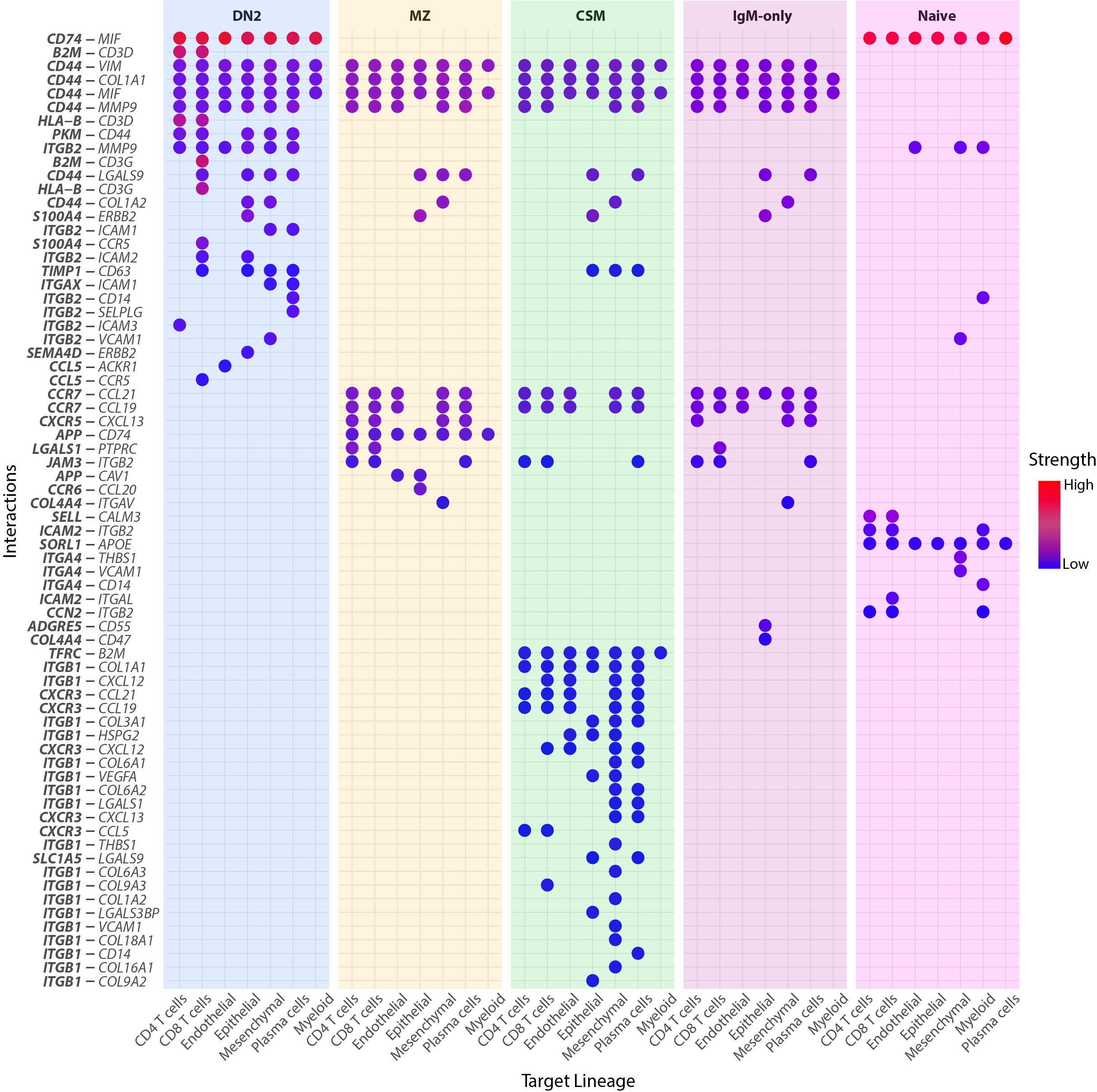


**Fig. S5. Imputed interactions associated with antigen presentation and cell migration and/or maintenance function of significant B cell marker genes across selected B cell subsets**

Dot plot showing the strength of imputed ligand-receptor interactions related to Antigen Presentation and Cell Migration and/or Maintenance involving B cell marker genes across five B cell subsets (DN2, MZ, CSM, IgM-only, and Naive). Dot color indicates interaction strength, with warmer colors representing stronger interactions.

**
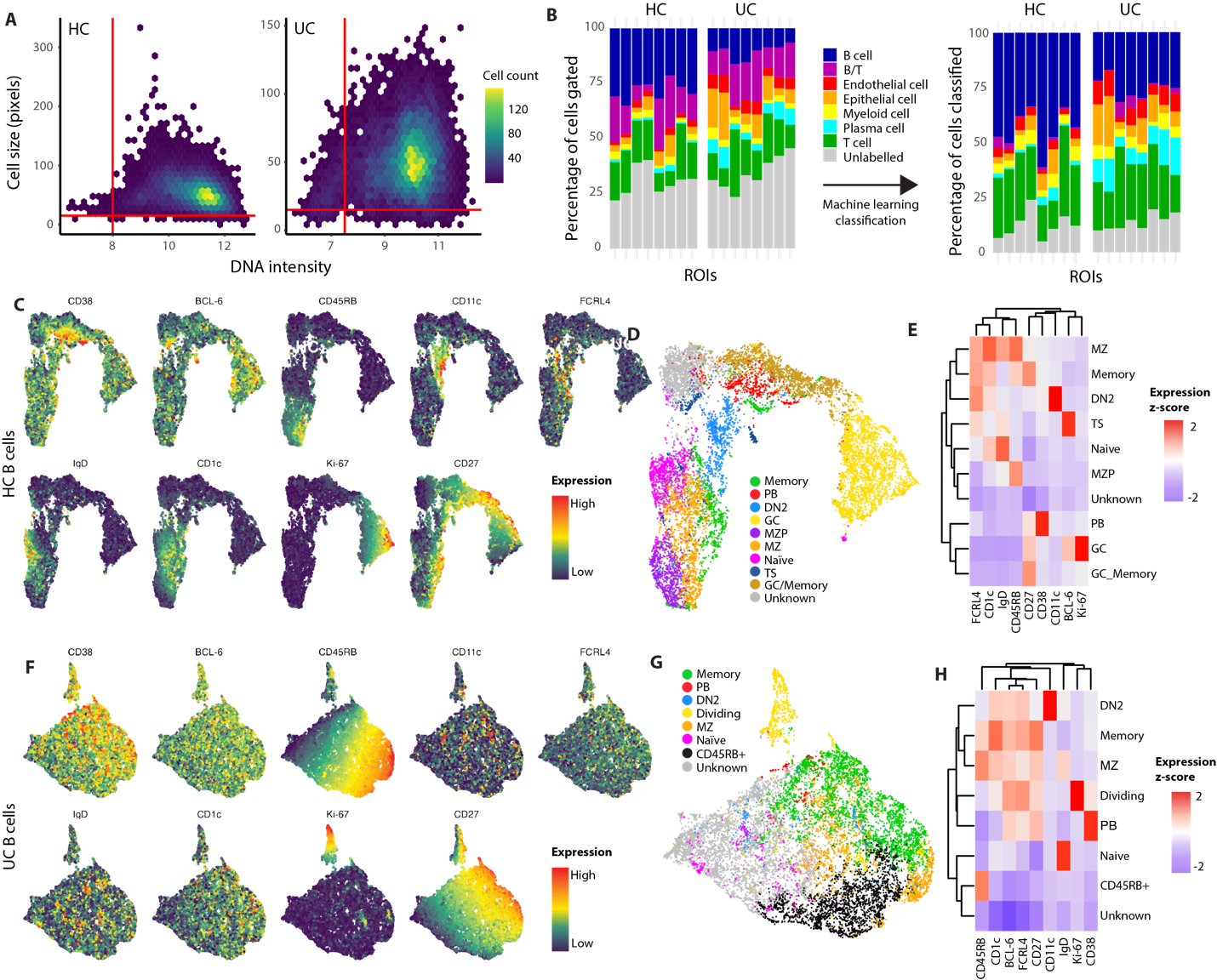
**

**Fig. S6.** **Computational comparisons of GALT cells from ulcerative colitis patients and healthy controls**

(A) Quality control of imaging mass cytometry data showing removal of segmented cells with low cell area and low DNA intensity (cell fragments). (B) B cell subsets classified in IMC data by manual gating (left) and subsequent machine learning classification (right). (C) UMAP showing expression of B cell subset markers in healthy appendix B cells in IMC. (D) UMAP showing B cell subset classification in healthy appendix B cells in IMC. (E) Heatmap showing expression of B cell subset markers in B cell subsets in healthy appendix B cells in IMC. (F) UMAP showing expression of B cell subset markers in UC appendix B cells in IMC. (G) UMAP showing B cell subset classification in UC appendix B cells in IMC. (H) Heatmap showing expression of B cell subset markers in B cell subsets in UC appendix B cells in IMC.

| **Table S1 Antibodies used in CITE-seq experiment** | | | |  |
| --- | --- | --- | --- | --- |
|  |  |  |  |  |

| **ADT marker** | **Clone** | **Supplier** | **1/ dilution factor** |
| --- | --- | --- | --- |
| ADT_IgD_TotalSeqC | IA6-2 | Biolegend | 50 |
| ADT_IgM_TotalSeqC | MHM-88 | Biolegend | 50 |
| ADT_CD10_TotalSeqC | HI10a | Biolegend | 50 |
| ADT_CD21_TotalSeqC | Bu32 | Biolegend | 50 |
| ADT_CD1C_TotalSeqC | L161 | Biolegend | 50 |
| ADT_CD27_TotalSeqC | Clone_323 | Biolegend | 50 |
| ADT_CD11C_TotalSeqC | S-HCL-3 | Biolegend | 50 |
| ADT_CXCR5_TotalSeqC | J252D4 | Biolegend | 50 |
| ADT_FCRL4_TotalSeqC | 413D2 | Biolegend | 50 |
| ADT_CD45RB_TotalSeqC | MEM-55 | Biolegend | 50 |

| **Antibody target** | **Fluorophore** | **Clone** | **Catalogue Number** | **Supplier** | **1/dilution factor** |
| --- | --- | --- | --- | --- | --- |
| CD19 | PE-Dazzle | SJ25C1 | 363031 | Biolegend | 50 |

| **Table S2 Antibodies used for immunofluorescence /confocal validation of interactions** | | | |  |
| --- | --- | --- | --- | --- |
|  |  |  |  |  |

| **Antibodies** | **Catalogue Number** | **Manufacturer** | **Species reactivity** | **1/dilution factor** |
| --- | --- | --- | --- | --- |
| B7-2 (D-6) (CD86) | sc-28347 | Santa Cruz | Mouse | 50 |
| Granulin | ab208777 | Abcam | Rabbit | 250 |
| EBI3 | ab307195 | Abcam | Rabbit | 250 |
| CD20 | ab244336 | Abcam | Rabbit | 1000 |
| CD20 | 382802 | BioLegend | Mouse | 50 |
| Donkey anti-Mouse IgG (H+L) Alexa Fluor™ Plus 555 - A32773 | A32773 | Thermo Fisher | Donkey | 500 |
| Donkey anti-Rabbit IgG (H+L) Alexa Fluor™ Plus 555 - A32794 | A32794 | Thermo Fisher | Donkey | 500 |
| F(ab')2-Goat anti-Rabbit IgG (H+L) Alexa Fluor™ Plus 488 - A48282TR | A48282TR | Thermo Fisher | Goat | 500 |
| F(ab')2-Goat anti-Mouse IgG (H+L) Alexa Fluor™ Plus 488 - A48286TR | A48286TR | Thermo Fisher | Goat | 500 |

**Table S3 Sample information**

**Appendix samples used for CITE-seq:**

| **Brain death donor (DBD) or cardiac death donor (DCD)** | **Disease** | **Age** | **Sex** |
| --- | --- | --- | --- |
| DBD | Healthy | 77 | F |
| DBD | Healthy | 70 | F |
| DCD | Healthy | 60 | M |

**Patients with UC who underwent right hemicolectomy**

| **Age at surgery** | **Sex** | **Disease duration (years)** | **Medication on admission** |
| --- | --- | --- | --- |
| 30 | M | 4 | azathioprine, humira |
| 28 | M | 1 | azathioprine |
| 47 | F | 8 | mesalasine |
| 27 | F | 16 | mesalasine |

In addition, twelve FFPE samples of normal human appendix were used for COSMX, for validation of imputed cellular interactions, and controls for comparison with UC appendix. No donor information is available for these samples.
